# Supplementary material for: Threats of global warming to the world’s freshwater fishes
Source: Nat Commun. 2021 Mar 15;12:1701. doi: 10.1038/s41467-021-21655-w (PMC7960982; doi:10.1038/s41467-021-21655-w)
Supplement: Supplementary file 1 — Supplementary Information [file 41467_2021_21655_MOESM1_ESM.pdf]

Supplementary Information for

Threats of global warming to the world's freshwater fishes

Valerio Barbarossa[a,b,c,*], Joyce Bosmans[b], Niko Wanders[d], Henry King[e], Marc F.P. Bierkens[d,f], Mark A.J. Huijbregts[b], and Aafke M. Schipper[b,c]

[a]Leiden University, Institute of Environmental Sciences (CML), Leiden, The Netherlands; [b]Radboud University, Department of Environmental Science, The Netherlands; [c]PBL Netherlands Environmental Assessment Agency, Department of Nature and Rural Areas, The Netherlands; [d]Utrecht University, Department of Physical Geography, The Netherlands; [e]Unilever R&D, Safety and Environmental Assurance Centre, UK; [f]Deltares, Utrecht, The Netherlands

*To whom correspondence may be addressed. Email: v.barbarossa@cml.leidenuniv.nl

This PDF file includes:

Supplementary Methods
Supplementary Supplementary Figures 1 to 13
Supplementary Supplementary Tables 1 to 6
Supplementary References

Supplementary Methods

Representativeness of species richness

We compared fish species richness, as derived from the dataset compiled in this study (N = 12,934), against a recent collection of freshwater fish species richness for the major world's watersheds[1] (Supplementary Figure 6). Tedesco et al.[1] provide a list of 14,953 freshwater fish species occurring in 3,119 watersheds, collected from both literature and web-based sources. Using the same watershed boundaries, we sampled the species of our database occurring in each watershed.

Validation of the water temperature threshold

We compared the species-specific maximum weekly water temperature thresholds derived in this study to laboratory data of critical thermal maxima (*CTmax*) for 409 species, compiled from a combination of sources[2,3]. This allowed us to check whether the threshold inferred from spatial data was representative of the actual thermal tolerance of freshwater fish species. For those species for which multiple experiments to quantify the thermal limit were reported, we averaged over the reported outcomes. We then compared such data to the maximum weekly water temperature threshold calculated in our study. Since the threshold is calculated for each historical period forced by a different GCM, we considered the median of the five values and reported the range in terms of maximum and minimum values across the GCM ensemble. To test the goodness of fit between inferred and lab data, we calculated the Pearson's correlation coefficient and the mean percent difference as $\sum_N \frac{|CTmax_{lab} - CTmax_{inferred}|}{CTmax_{lab}} / N * 100$.

Details on the GCM-RCP ensemble

For the climate forcing applied to the hydrological model PCR-GLOBWB, we employed the five global climate models (GCMs) available from the InterSectoral Impact Model Intercomparison Project (ISI-MIP)[4]. The GCMs used are HadGEM2-ES from the Met-Office Hadley Centre, IPSL-CM5A-LR from the Institute Pierre Simon Laplace Climate Modelling Centre, MIROC-ESM-CHEM from a Japanese consortium (University of Tokio, NIES and JAMSTEC), GFDL-ESM2M from the Geophysical Fluid Dynamics Laboratory and NorESM1-M from a consortium of Norwegion universities and institutes. They were selected based on availability in the CMIP5 archive. ISI-MIP tailored GCM output for use in impact models by bias-correcting and spatially down-scaling the relevant climate variables[5,6]. The climate variables from the 5 GCMs used as input for the hydrological model PCR-GLOBWB[7] and water temperature model DynWAT[8] consisted of precipitation, temperature, reference potential evapotranspiration calculated with Penman-Monteith[9] and radiation components.

Supplementary Figures

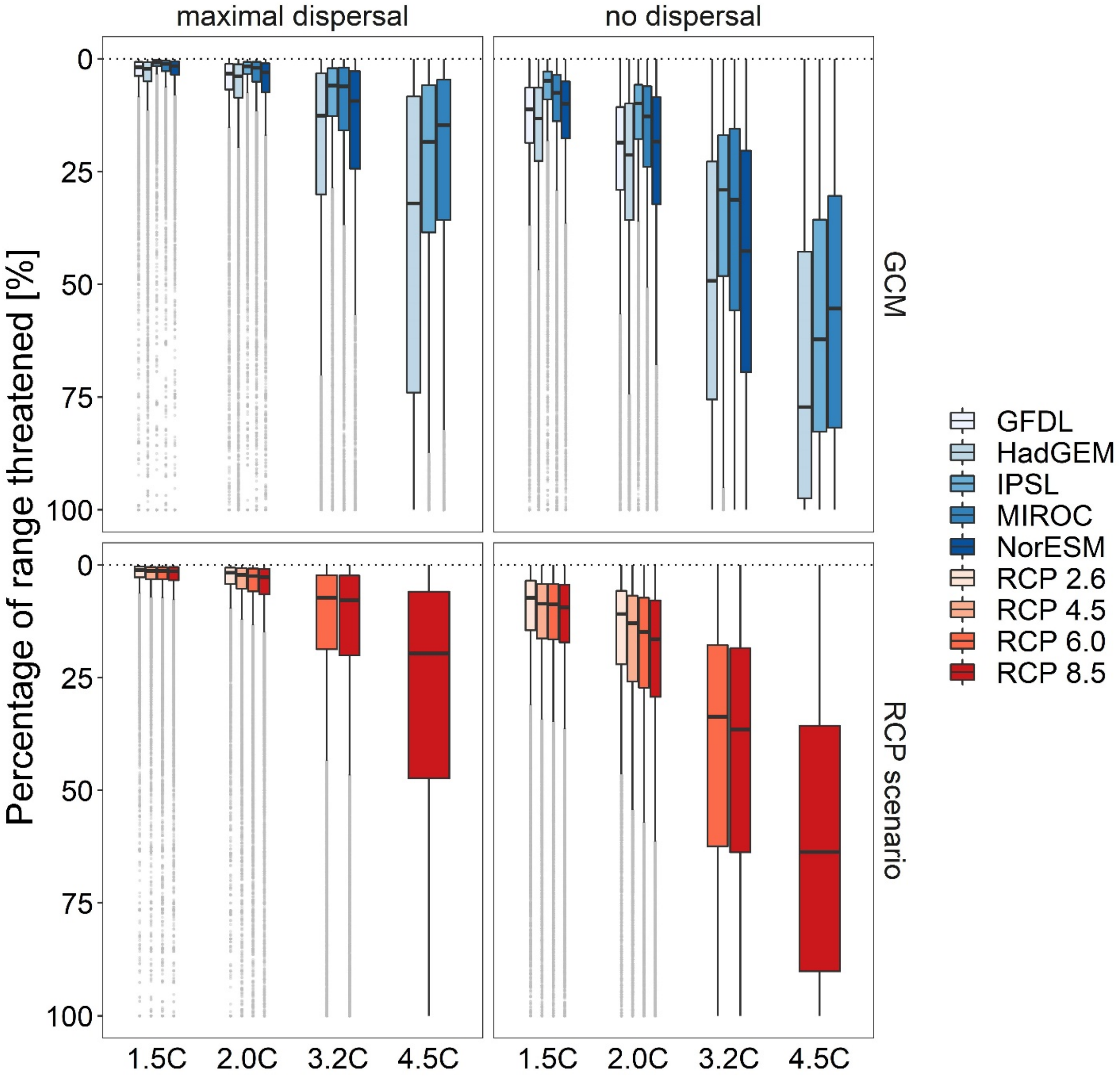

Supplementary Figure 1. Output variability stemming from the different inputs of Global Climate Models and Representative Concentration Pathway scenarios. Each box delimits the interquartile range and shows the median, and whiskers stand for the 95% interval for the 11,425 fish species assessed in this study.

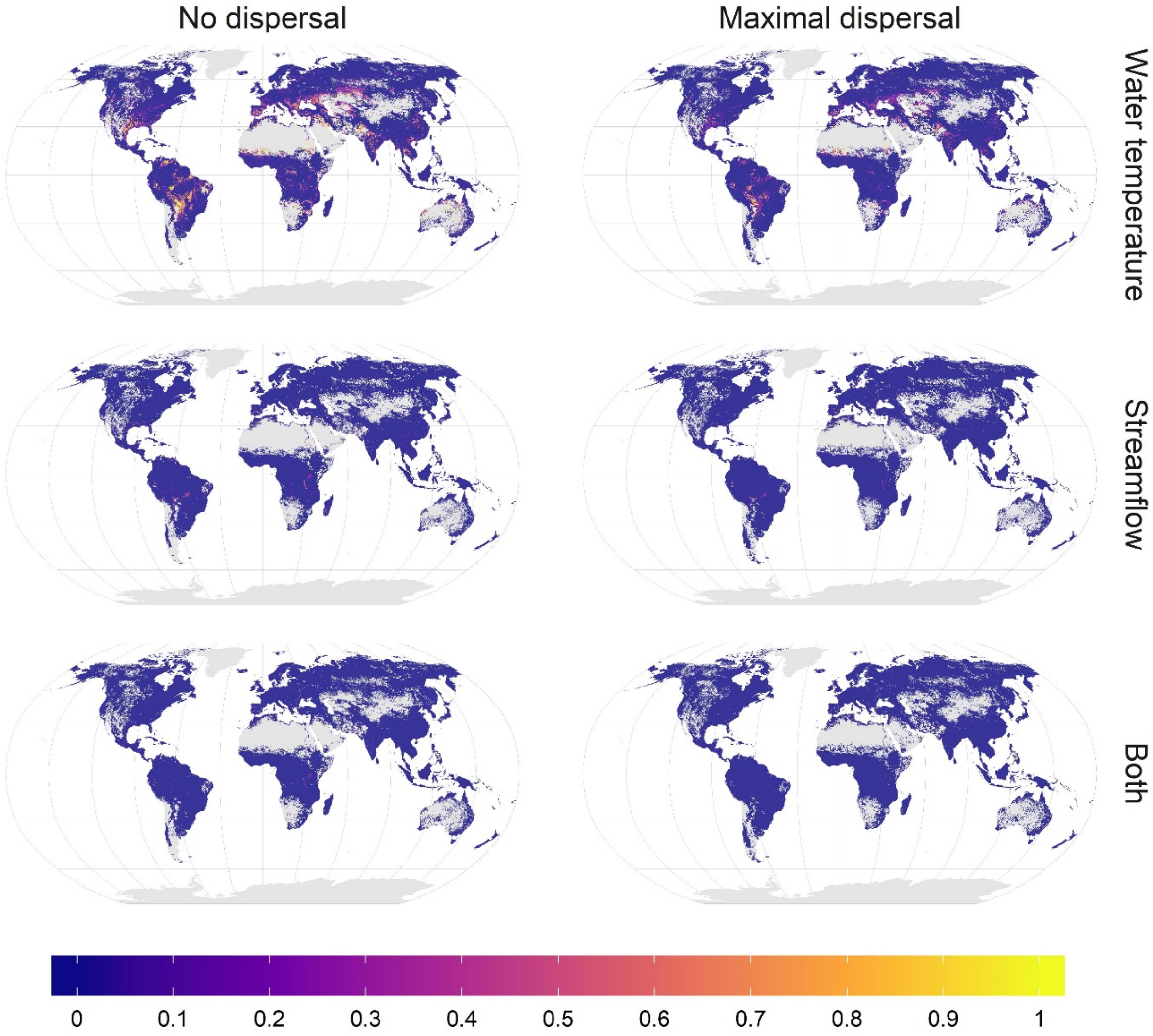

Supplementary Figure 2-I. Potentially affected fraction (PAF) of species due to changes in water temperature (top), flow conditions (center) or both (bottom) for the 1.5^{o}C warming scenario. The maps represent the median proportion of species affected over the GCM-RCP combinations available for the 1.5^{o}C warming scenario. Gray denotes no data areas (no species occurring or no data available). Source data are provided as Supplementary Data 2-3.

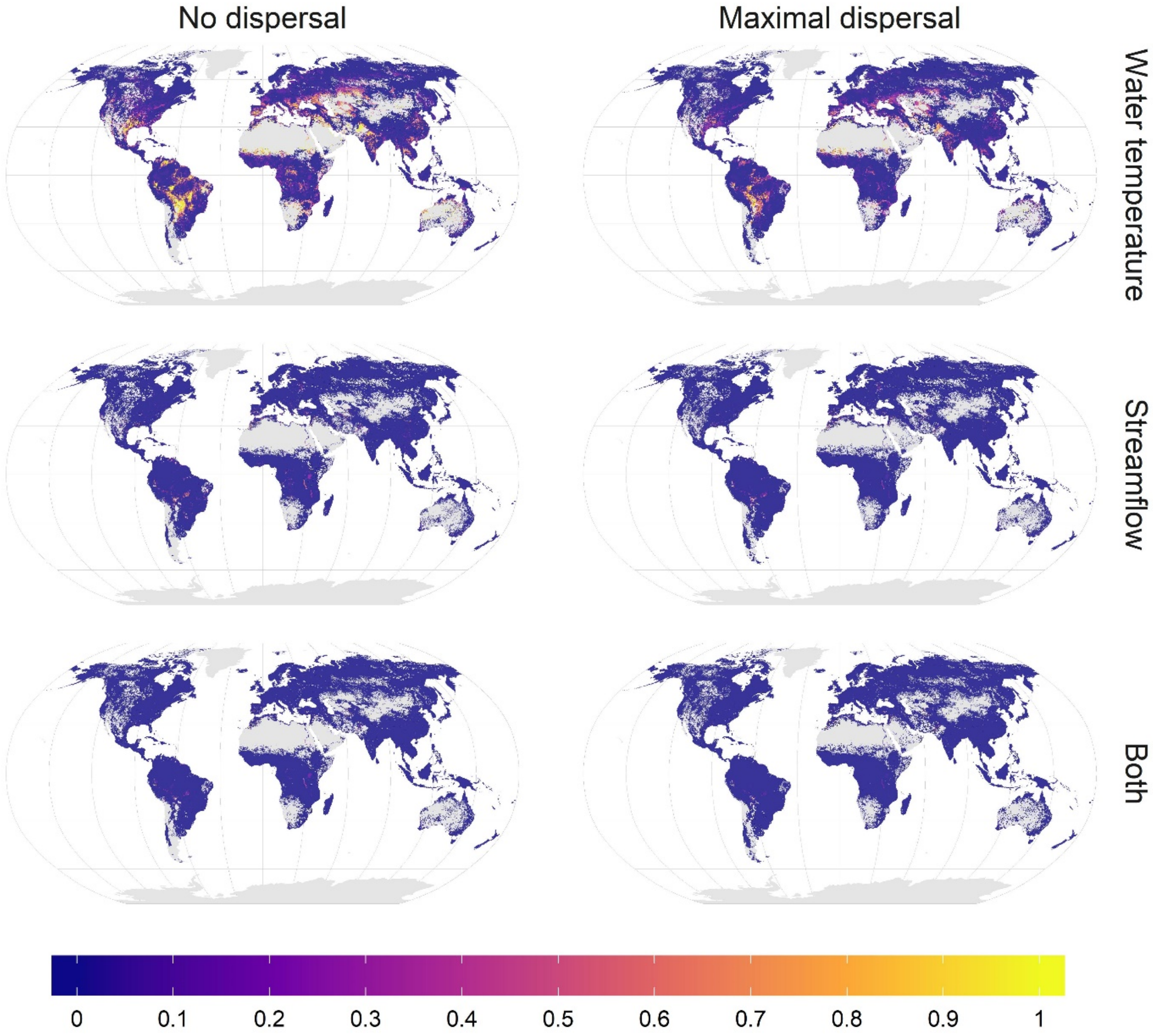

Supplementary Figure 2-II. Potentially affected fraction (PAF) of species due to changes in water temperature (top), flow conditions (center) or both (bottom) for the 2°C warming scenario. The maps represent the median proportion of species affected over the GCM-RCP combinations available for the 2°C warming scenario. Gray denotes no data areas (no species occurring or no data available). Source data are provided as Supplementary Data 2-3.

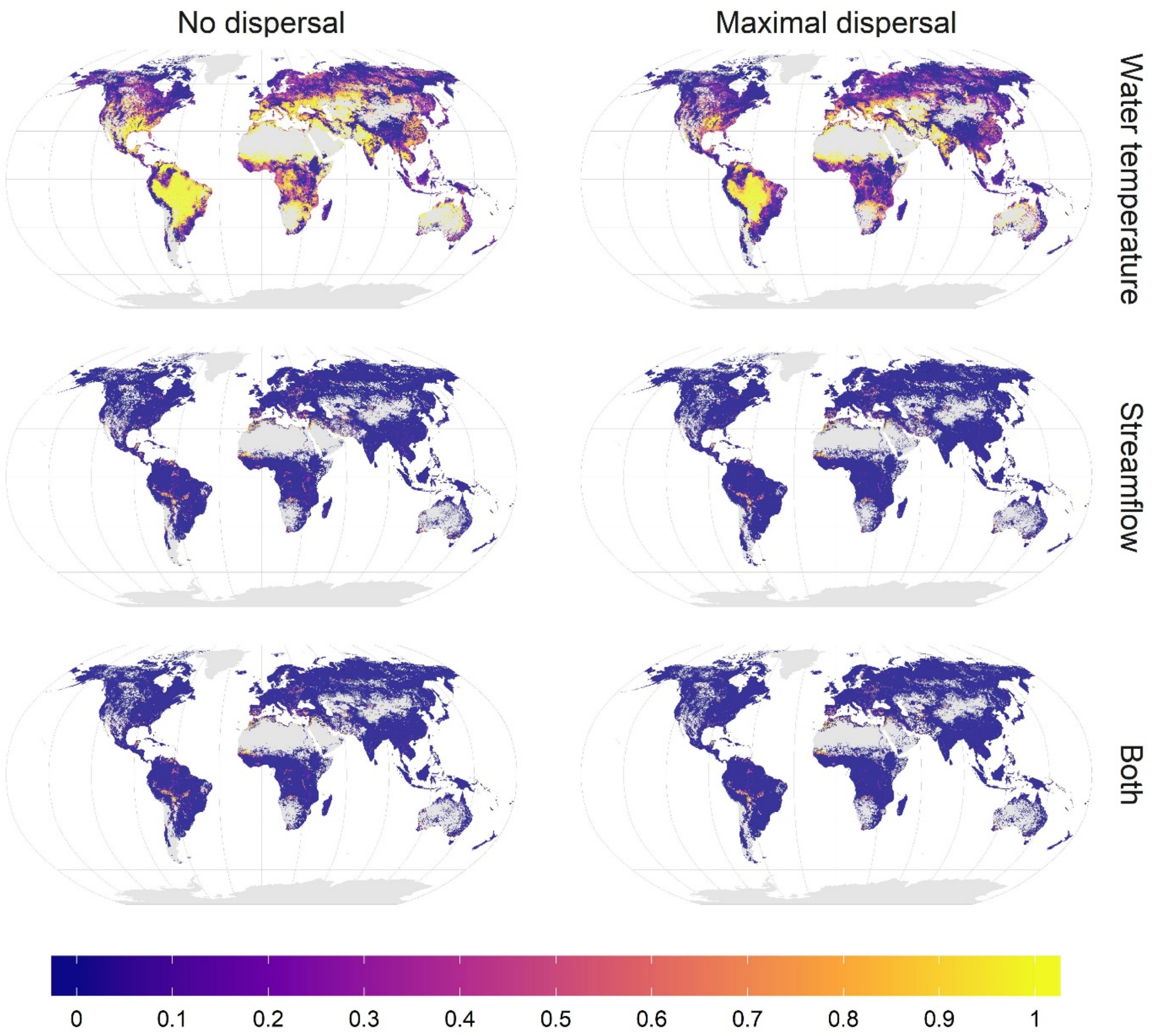

Supplementary Figure 2-III. Potentially affected fraction (PAF) of species due to changes in water temperature (top), flow conditions (center) or both (bottom) for the 4.5°C warming scenario. The maps represent the median proportion of species affected over the GCM-RCP combinations available for the 4.5°C warming scenario. Gray denotes no data areas (no species occurring or no data available). Source data are provided as Supplementary Data 2-3.

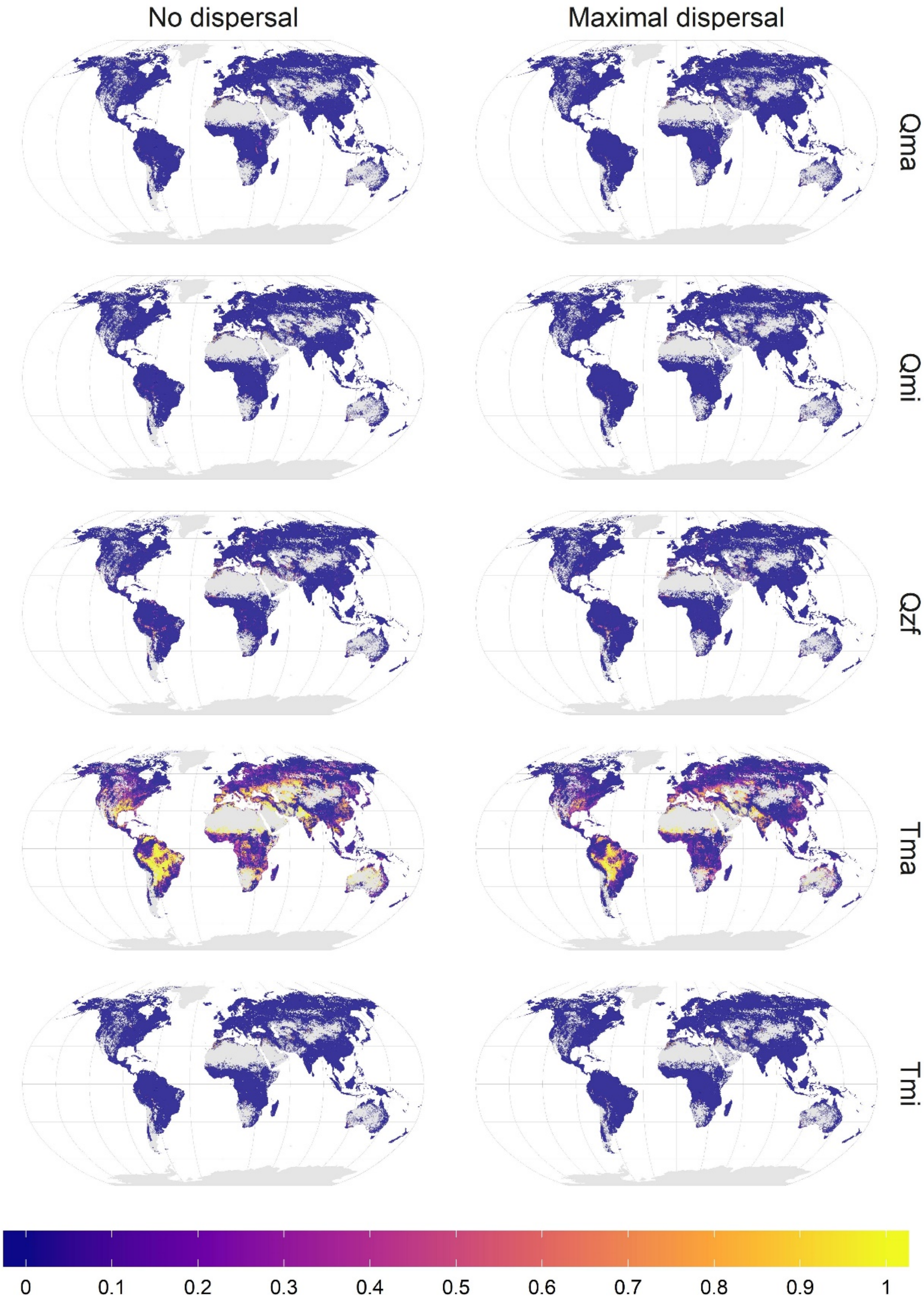

Supplementary Figure 3. Potentially affected fraction (PAF) of species due to changes in maximum flow (Qma), minimum flow (Qmi), number of zero flow weeks (Qzf), maximum water temperature (Tma) and minimum water temperature (Tmi) for the 3.2°C warming scenario. The maps represent the median proportion of species affected over the GCM-RCP combinations available for the 3.2°C scenario. Gray denotes no data areas (no species occurring or no data available). Source data are provided as Supplementary Data 2-3.

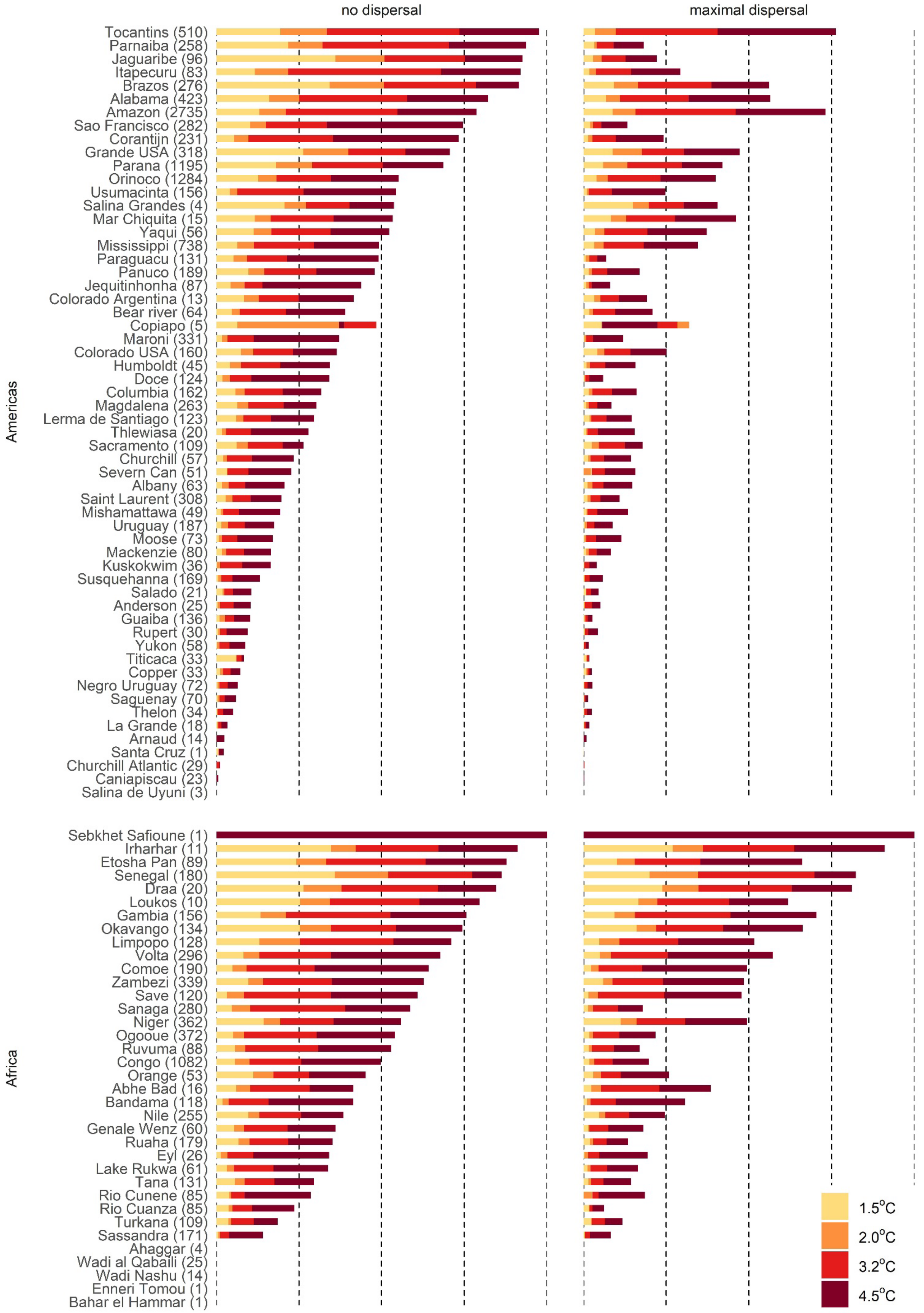
no dispersal
maximal dispersal
Americas
Tocantins (510)
Parnaiba (258)
Jaguaribe (96)
Itapecuru (83)
Brazos (276)
Alabama (423)
Amazon (2735)
Sao Francisco (282)
Corantijn (231)
Grande USA (318)
Parana (1195)
Orinoco (1284)
Usumacinta (156)
Salina Grandes (4)
Mar Chiquita (15)
Yaqui (56)
Mississippi (738)
Paraguacu (131)
Panuco (189)
Jequitinhonha (87)
Colorado Argentina (13)
Bear river (64)
Copiapo (5)
Maroni (331)
Colorado USA (160)
Humboldt (45)
Doce (124)
Columbia (162)
Magdalena (263)
Lerma de Santiago (123)
Thlewiasa (20)
Sacramento (109)
Churchill (57)
Severn Can (51)
Albany (63)
Saint Laurent (308)
Mishamattawa (49)
Uruguay (187)
Moose (73)
Mackenzie (80)
Kuskokwim (36)
Susquehanna (169)
Salado (21)
Anderson (25)
Guaiba (136)
Rupert (30)
Yukon (58)
Titicaca (33)
Copper (33)
Negro Uruguay (72)
Saguenay (70)
Thelon (34)
La Grande (18)
Arnaud (14)
Santa Cruz (1)
Churchill Atlantic (29)
Caniapiscau (23)
Salina de Uyuni (3)
Africa
Sebkhet Safioune (1)
Irharhar (11)
Etosha Pan (89)
Senegal (180)
Draa (20)
Loukos (10)
Gambia (156)
Okavango (134)
Limpopo (128)
Volta (296)
Comoe (190)
Zambezi (339)
Save (120)
Sanaga (280)
Niger (362)
Ogooue (372)
Ruvuma (88)
Congo (1082)
Orange (53)
Abhe Bad (16)
Bandama (118)
Nile (255)
Genale Wenz (60)
Ruaha (179)
Eyl (26)
Lake Rukwa (61)
Tana (131)
Rio Cunene (85)
Rio Cuanza (85)
Turkana (109)
Sassandra (171)
Ahaggar (4)
Wadi al Qabaili (25)
Wadi Nashu (14)
Enneri Tomou (1)
Bahar el Hammar (1)
1.5°C
2.0°C
3.2°C
4.5°C

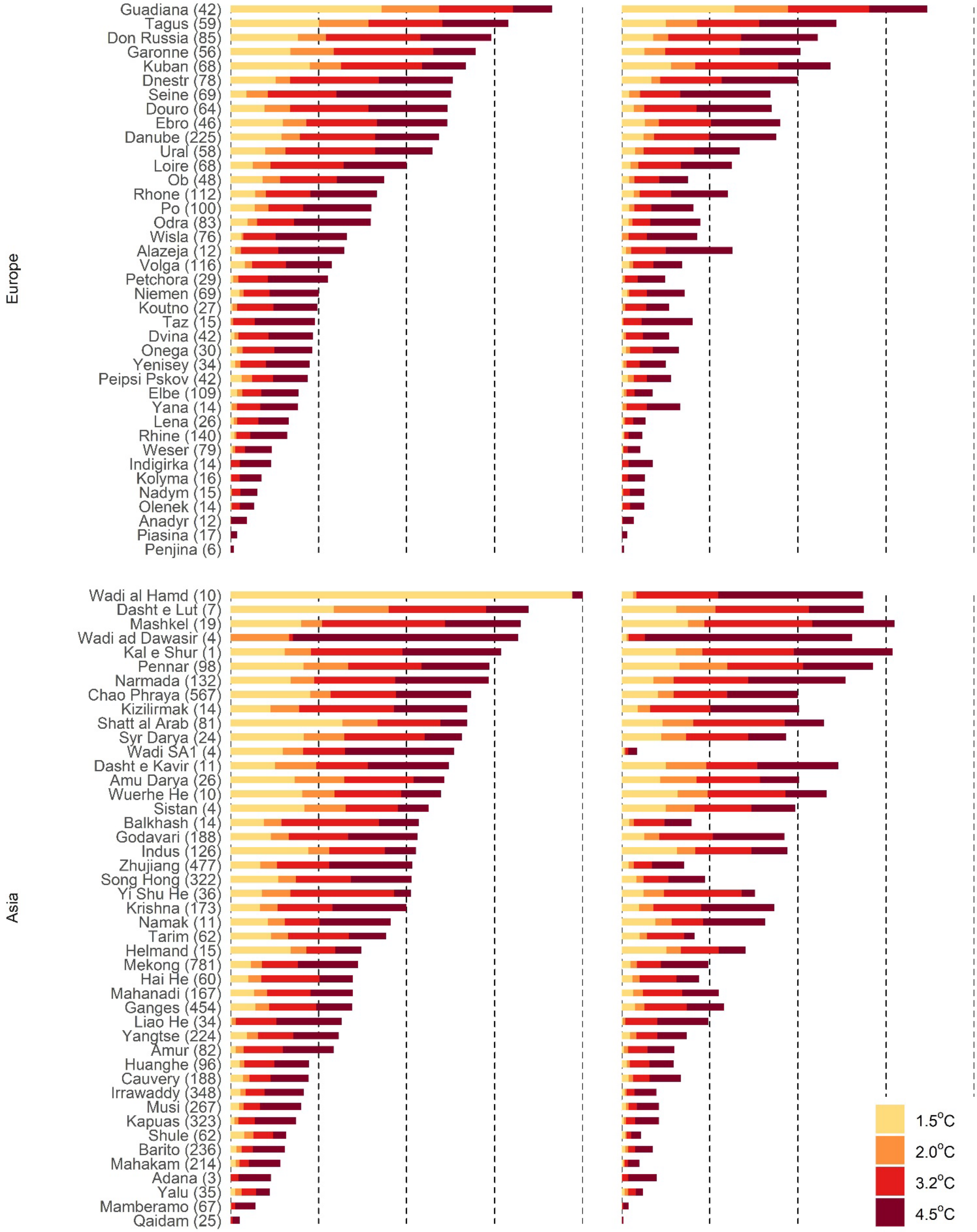
Europe
Guadiana (42)
Tagus (59)
Don Russia (85)
Garonne (56)
Kuban (68)
Dnestr (78)
Seine (69)
Douro (64)
Ebro (46)
Danube (225)
Ural (58)
Loire (68)
Ob (48)
Rhone (112)
Po (100)
Odra (83)
Wisla (76)
Alazeja (12)
Volga (116)
Petchora (29)
Niemen (69)
Koutno (27)
Taz (15)
Dvina (42)
Onega (30)
Yenisey (34)
Peipsi Pskov (42)
Elbe (109)
Yana (14)
Lena (26)
Rhine (140)
Weser (79)
Indigirka (14)
Kolyma (16)
Nadym (15)
Olenek (14)
Anadyr (12)
Piasina (17)
Penjina (6)
Asia
Wadi al Hamd (10)
Dasht e Lut (7)
Mashkel (19)
Wadi ad Dawasir (4)
Kal e Shur (1)
Pennar (98)
Narmada (132)
Chao Phraya (567)
Kizilirmak (14)
Shatt al Arab (81)
Syr Darya (24)
Wadi SA1 (4)
Dasht e Kavir (11)
Amu Darya (26)
Wuerhe He (10)
Sistan (4)
Balkhash (14)
Godavari (188)
Indus (126)
Zhujiang (477)
Song Hong (322)
Yi Shu He (36)
Krishna (173)
Namak (11)
Tarim (62)
Helmand (15)
Mekong (781)
Hai He (60)
Mahanadi (167)
Ganges (454)
Liao He (34)
Yangtse (224)
Amur (82)
Huanghe (96)
Cauvery (188)
Irrawaddy (348)
Musi (267)
Kapuas (323)
Shule (62)
Barito (236)
Mahakam (214)
Adana (3)
Yalu (35)
Mamberamo (67)
Qaidam (25)
1.5°C
2.0°C
3.2°C
4.5°C

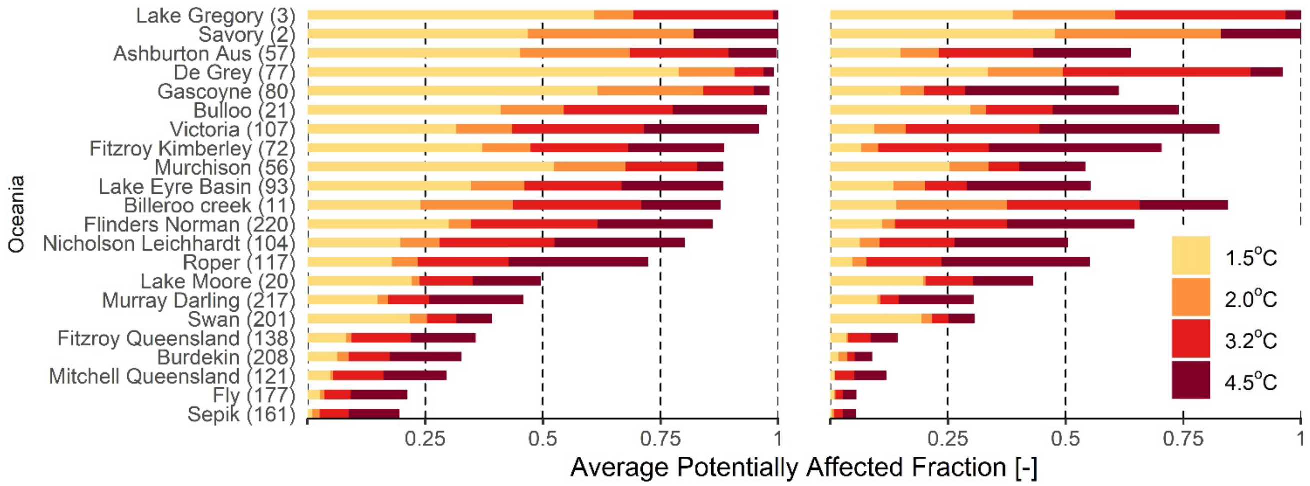

Supplementary Figure 4. Average potentially affected fraction over the 200 largest watersheds of the world (excluding watersheds with less than 25 species). Continents are defined according to the World Bank Development Indicators (www.worldbank.org). Numbers in brackets represent the number of species within the watershed. Source data are provided as Supplementary Data 4.

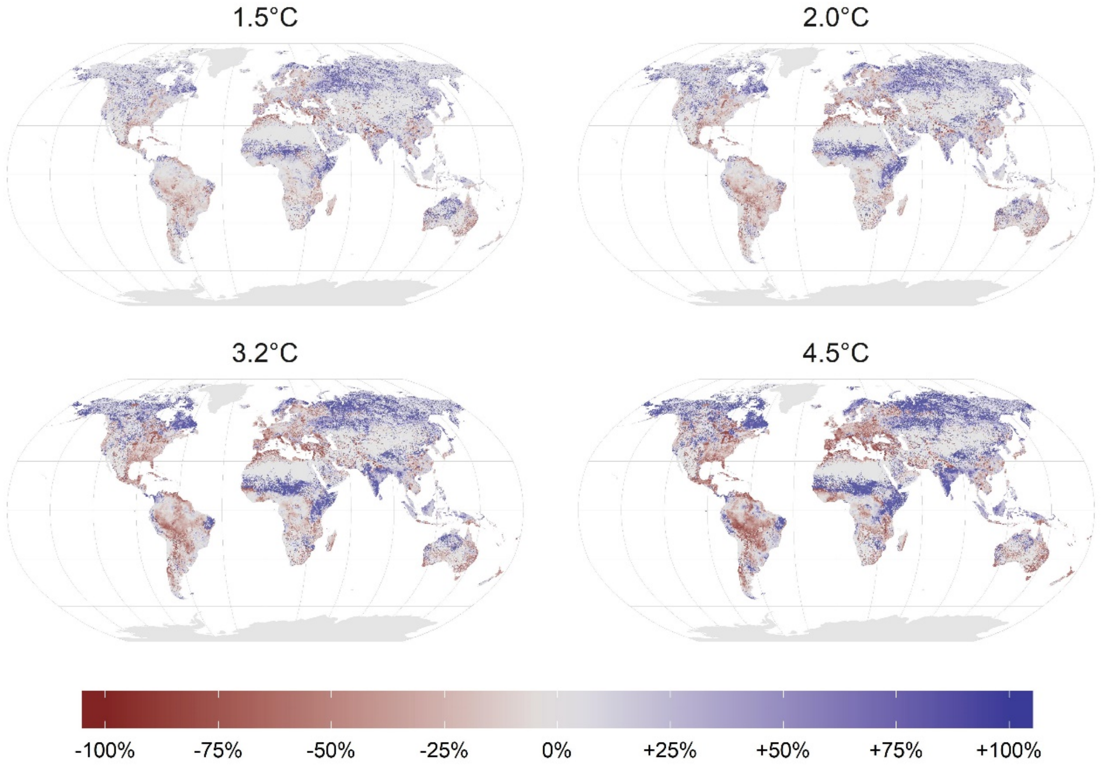

Supplementary Figure 5-I. Percentage of change in minimum weekly flow at the four different warming levels compared to the historical period (1976-2005). Percentage of change is expressed as (future - historical)/historical. Each map represents the median of the GCM-RCP ensemble at each warming level. For simplicity of representation, values >100% are set to 100%.

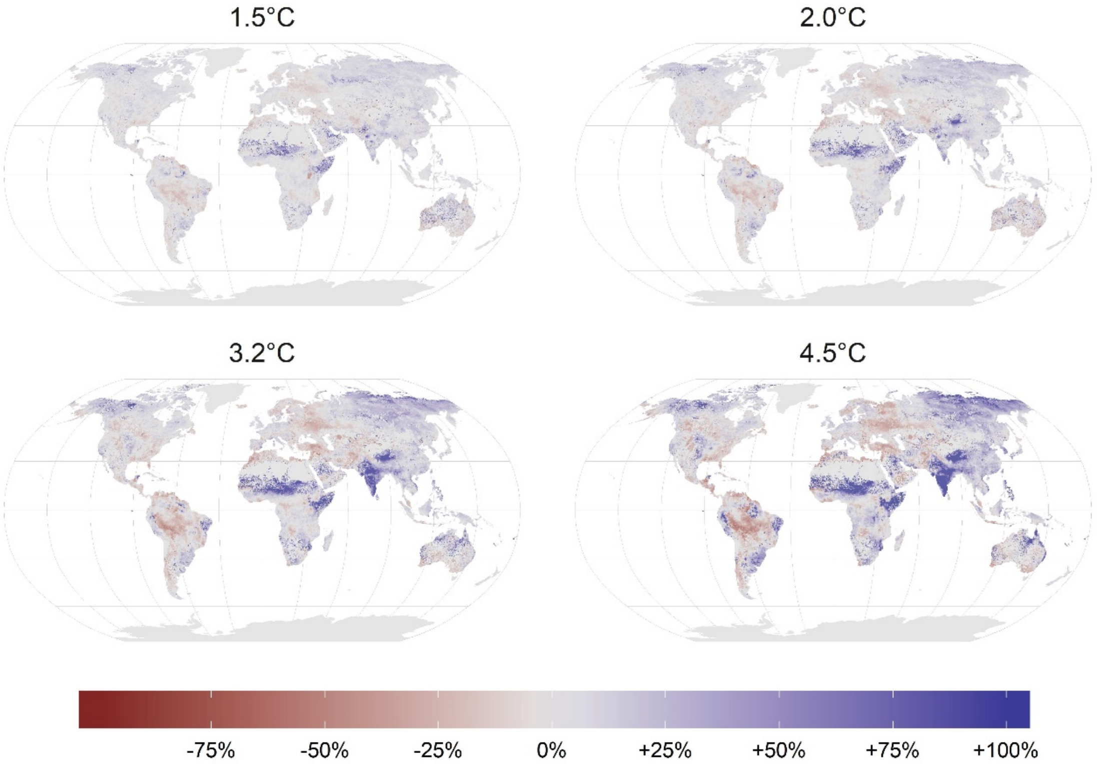

Supplementary Figure 5-II. Percentage of change in maximum weekly flow at the four different warming levels compared to the historical period (1976-2005). Percentage of change is expressed as (future - historical)/historical. Each map represents the median of the GCM-RCP ensemble at each warming level. For simplicity of representation, values >100% are set to 100%.

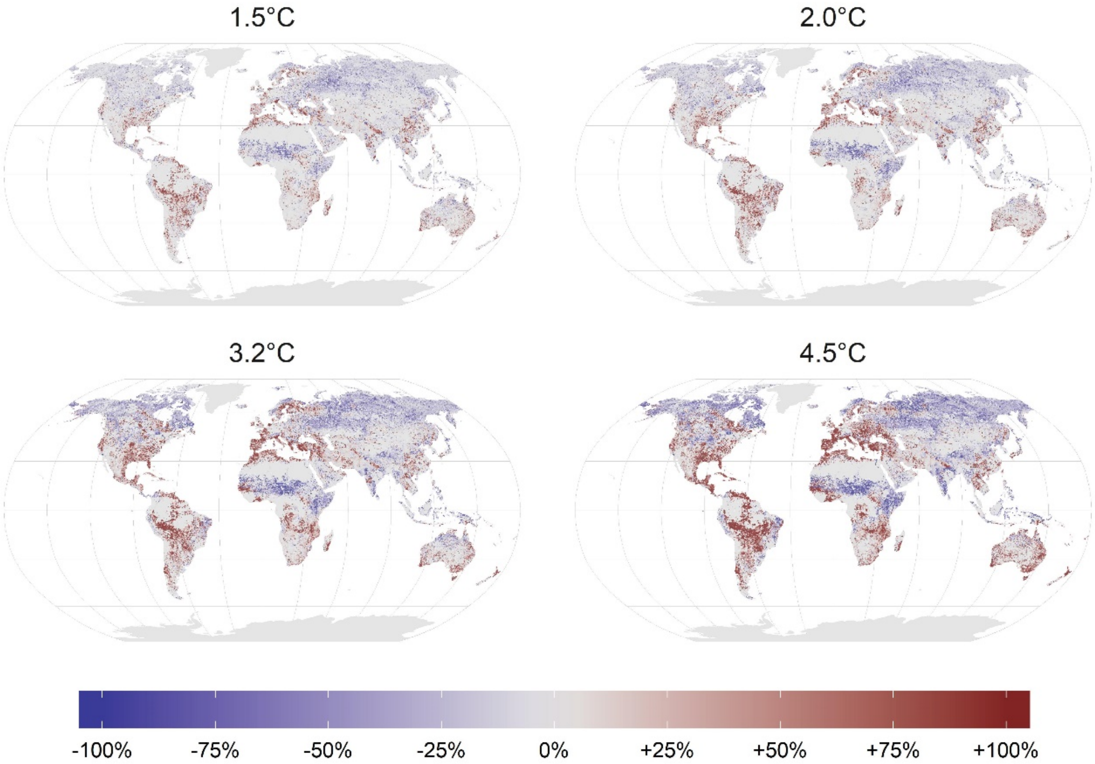

Supplementary Figure 5-III. Percentage of change in number of zero flow weeks at the four different warming levels compared to the historical period (1976-2005). Percentage of change is expressed as (future - historical)/historical. Each map represents the median of the GCM-RCP ensemble at each warming level. For simplicity of representation, values >100% are set to 100%.

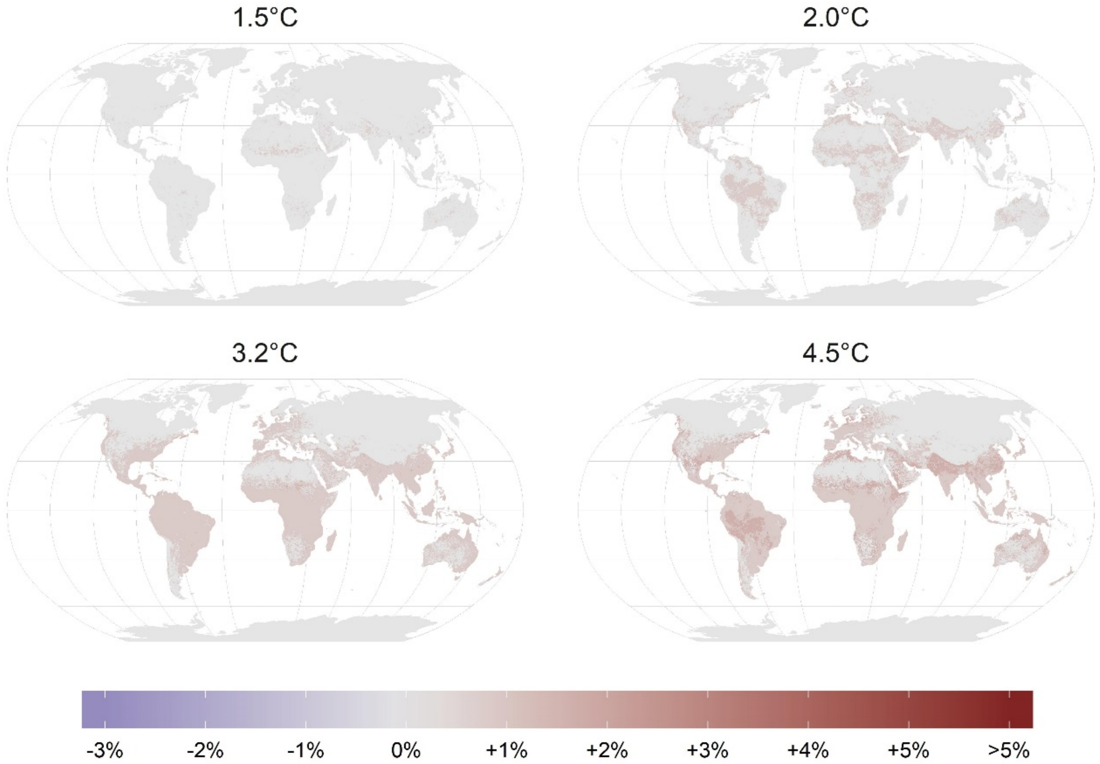

Supplementary Figure 5-IV. Percentage of change in minimum weekly water temperature at the four different warming levels compared to the historical period (1976-2005). Percentage of change is expressed as (future - historical)/historical. Each map represents the median of the GCM-RCP ensemble at each warming level.

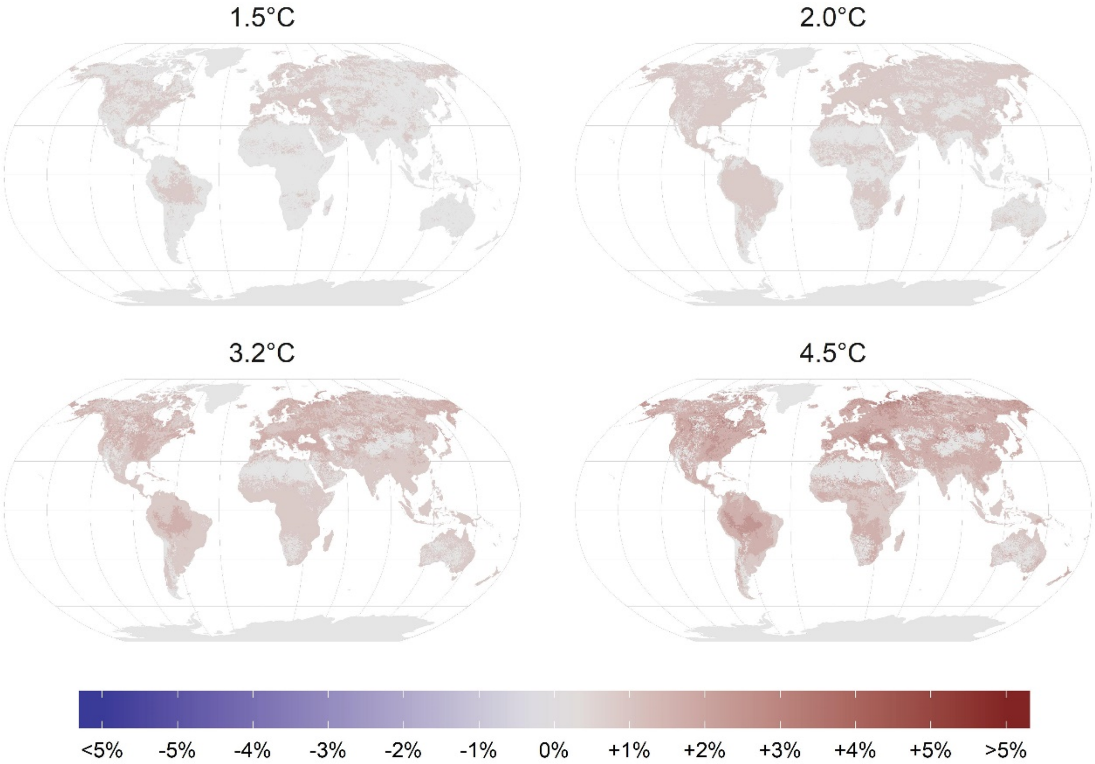

Supplementary Figure 5-V. Percentage of change in maximum weekly water temperature at the four different warming levels compared to the historical period (1976-2005). Percentage of change is expressed as (future - historical)/historical. Each map represents the median of the GCM-RCP ensemble at each warming level.

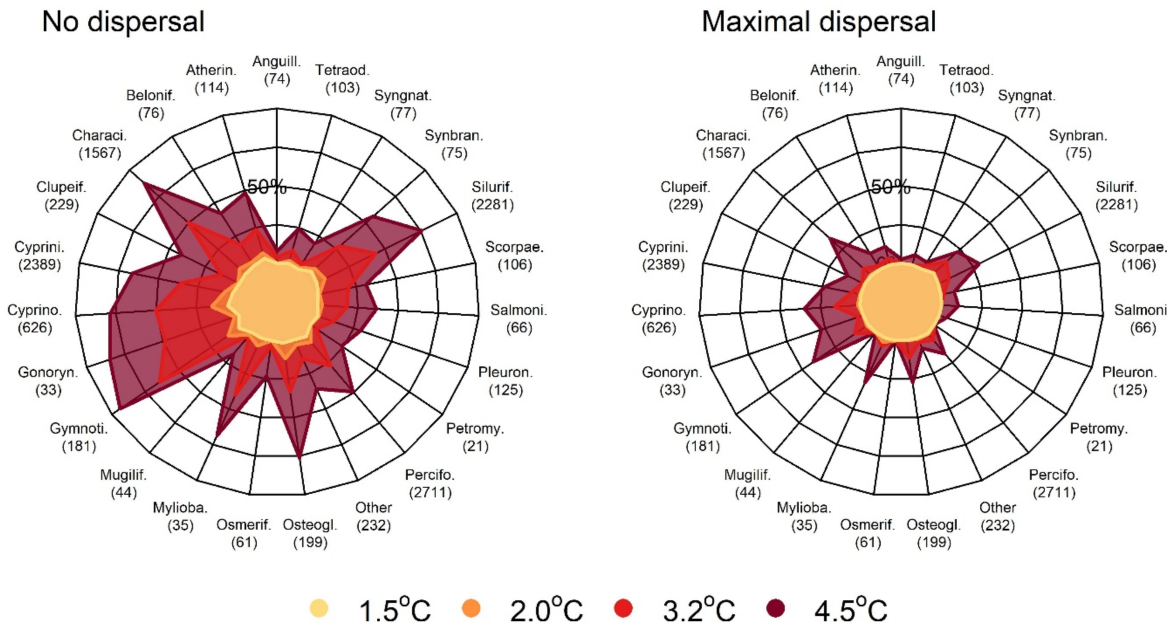

Supplementary Figure 6. Mean geographic range threatened summarized by species order. Groups with less than 20 species represented in our database are grouped together in the "other" category for simplicity of representation (the full list of order names is provided in Supplementary Table 2). The outer line represents 100% and each inner line is drawn with a 25% interval.

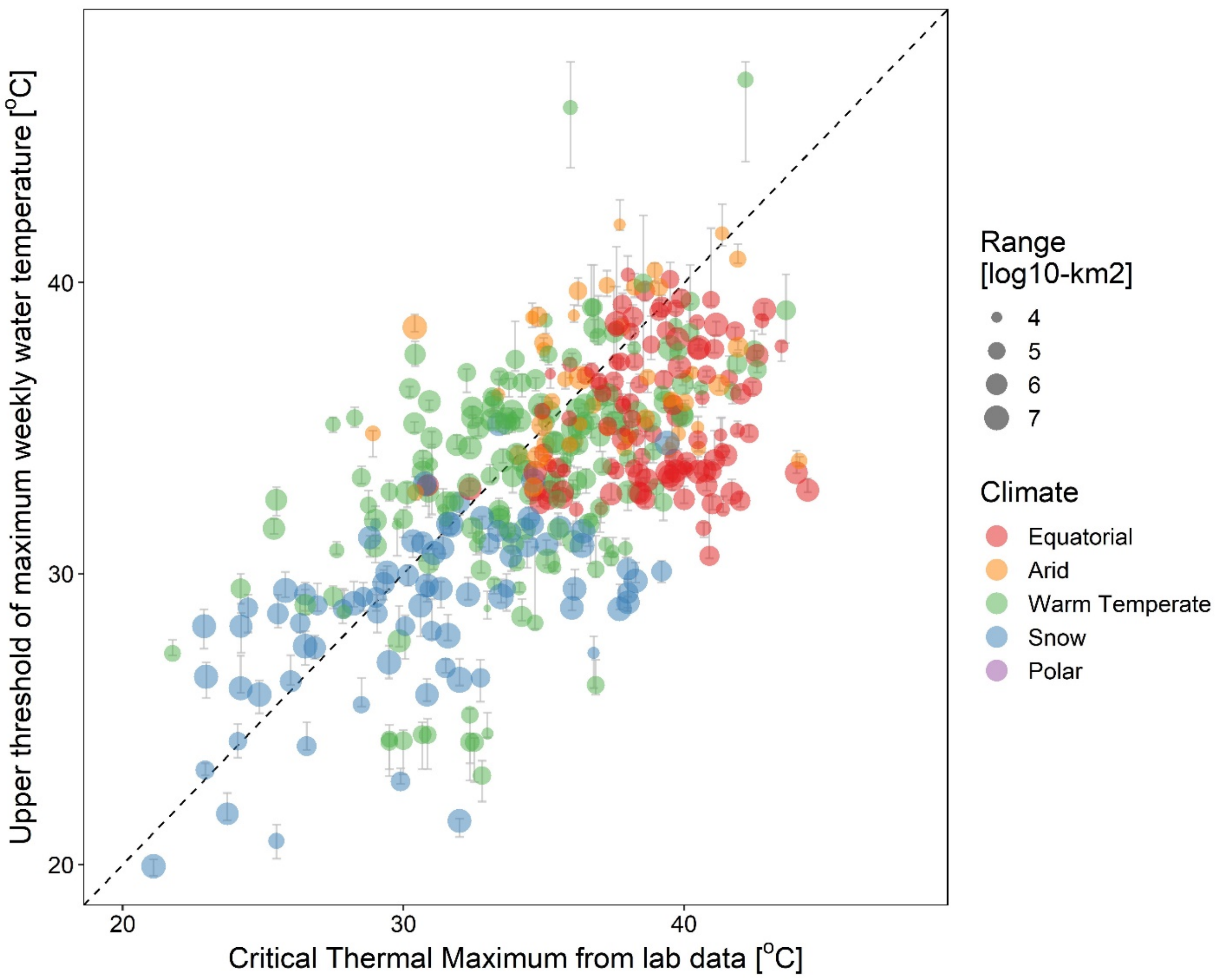

Supplementary Figure 7. Validation of the upper threshold of maximum weekly water temperature used in this study against critical thermal maxima from laboratory experiments (n = 409 species). The error bars represent full range of values from the five GCMs used in this study, while the dots show the median value. The dots size is proportional to the area of the occurrence range of each species and the values in the scale stands for a round of ±0.5. Climate zoning is according to the historic Köppen-Geiger classification. The 1:1 line is shown as a black dashed line. Mean percent difference = 9%; Peason's r = 0.62.

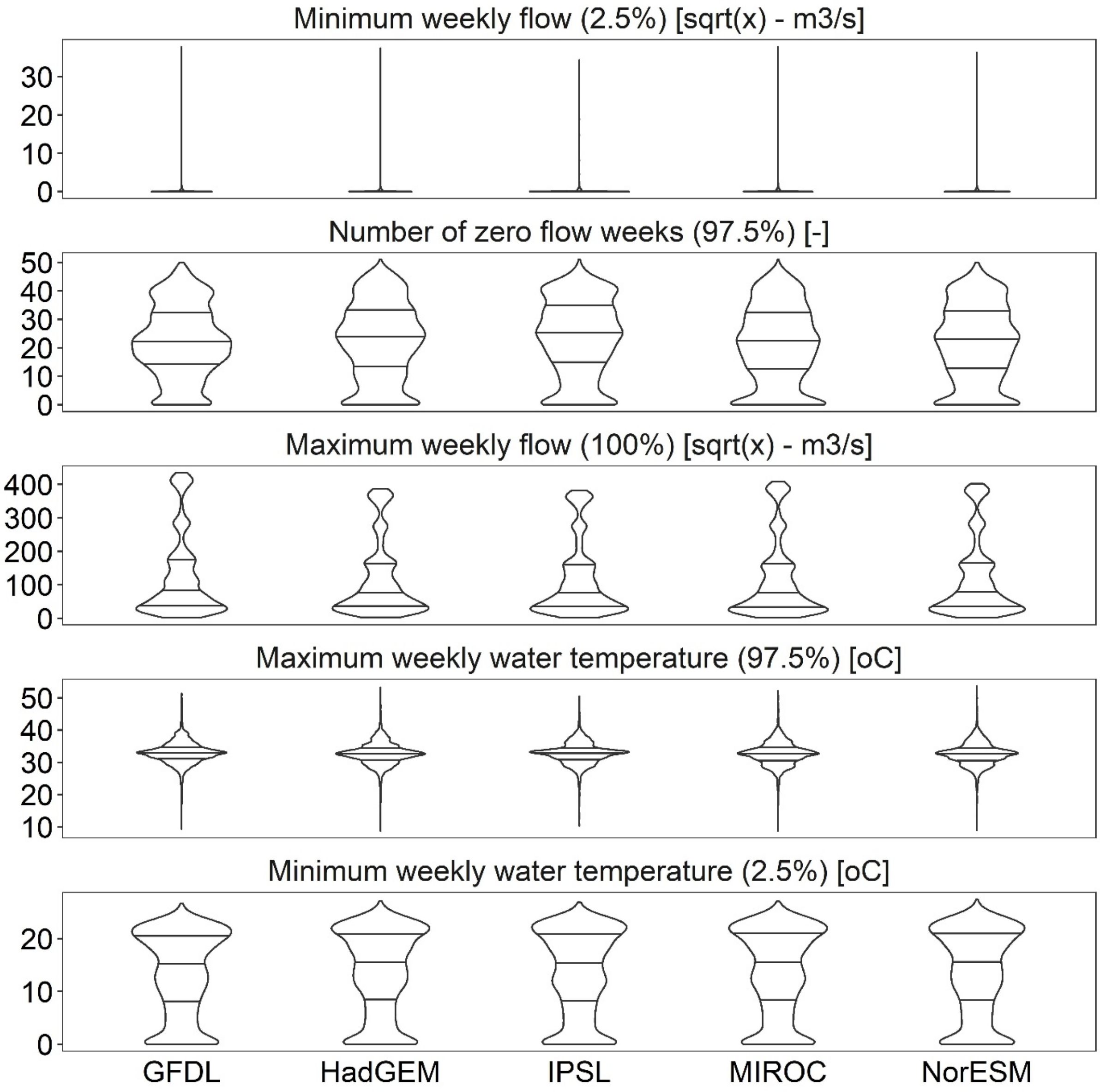

Supplementary Figure 8. Statistical distribution of the flow and water temperature thresholds used in this study for the 11,425 species, represented as violin plots. For each variable, the threshold is calculated for each GCM historical period 1976-2005. In brackets, the percentile used for each threshold. Horizontal lines represent the interquartile range and median.

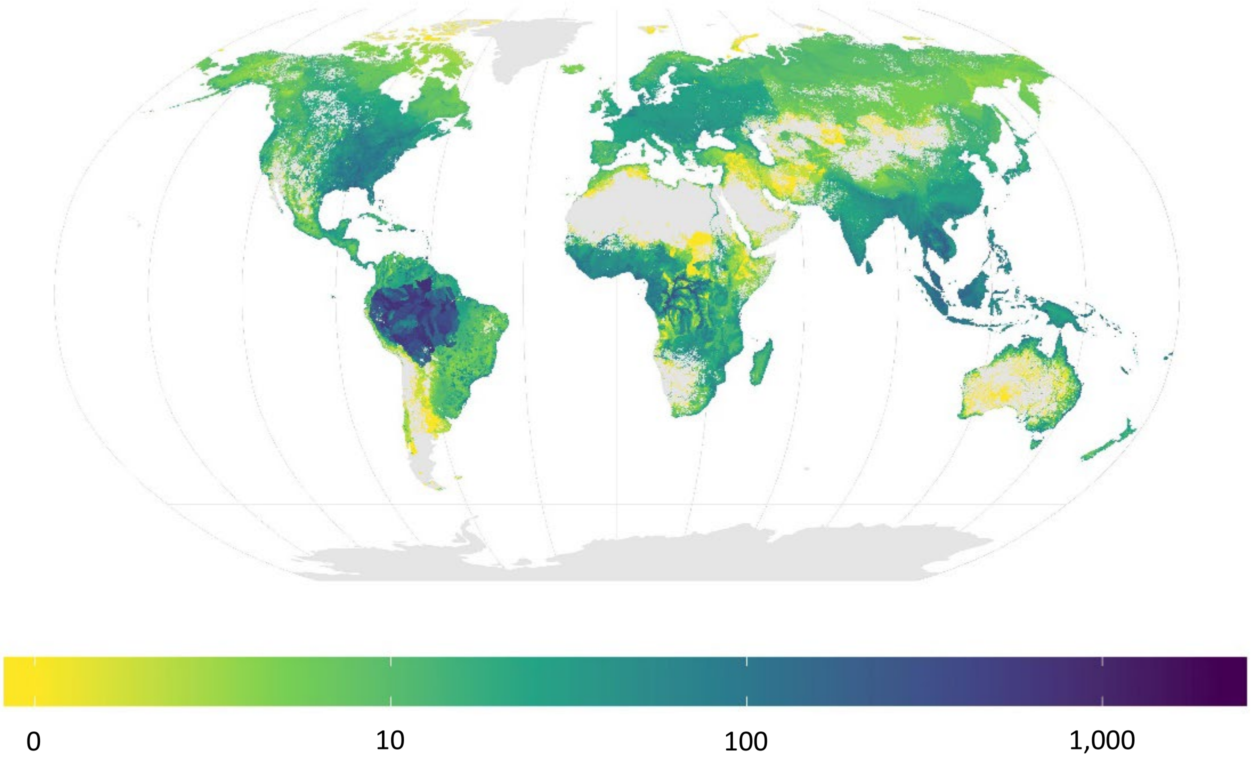

Supplementary Figure 9. Current species richness at each five arc-minutes grid cell derived from the geographic range maps for the 11,425 riverine fish species used in this study. Gray denotes no data. Source data are provided as Supplementary Data 6.

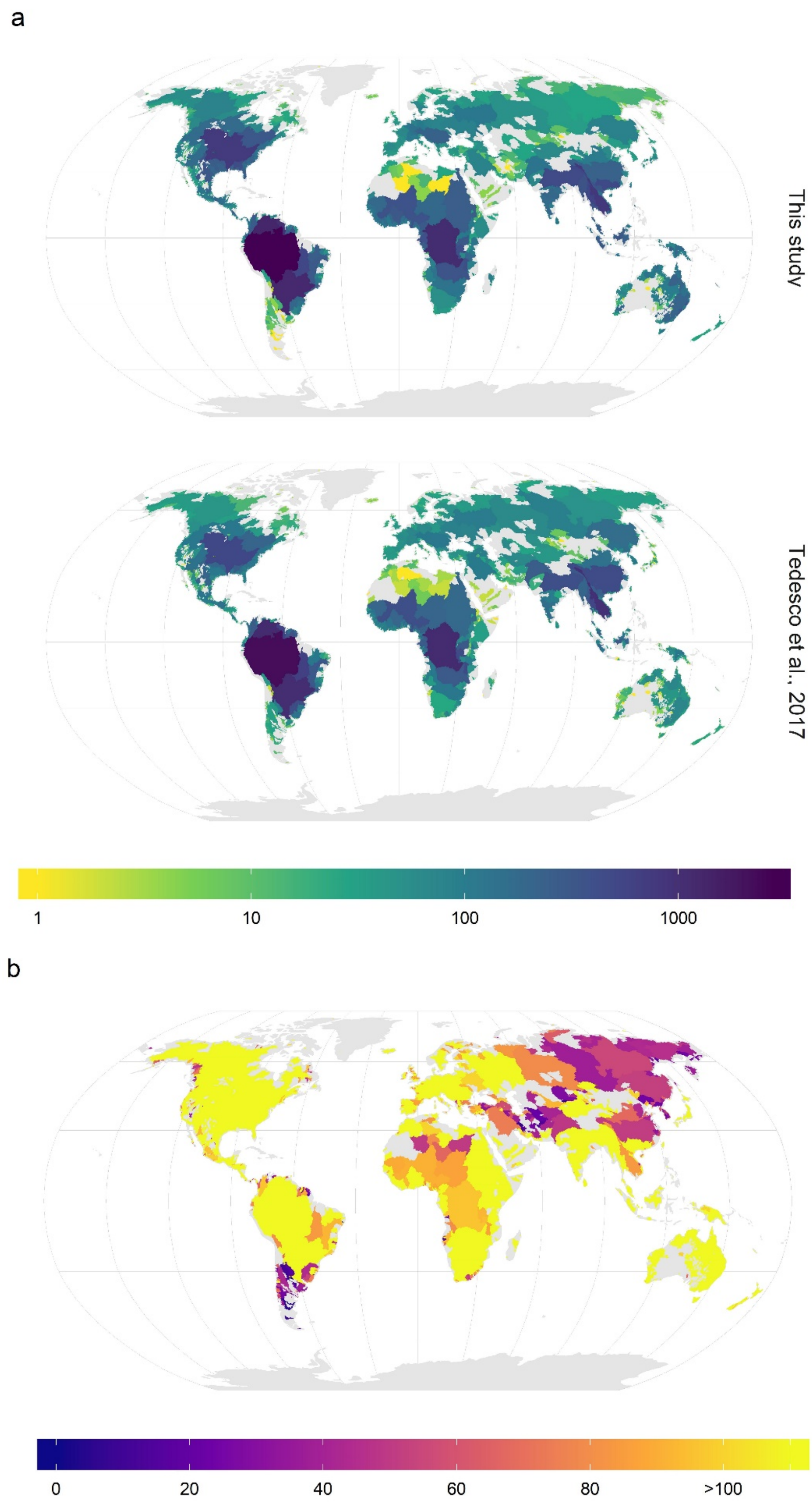

Supplementary Figure 10. Total number of species (a) and (b) of freshwater fish species covered by the data used in this study compared to Tedesco et al.[1] for the major watersheds of the world (as available in Tedesco et al.[1], geographic range coverage of this study is provided in Supplementary Figure 9). Gray denotes no data.

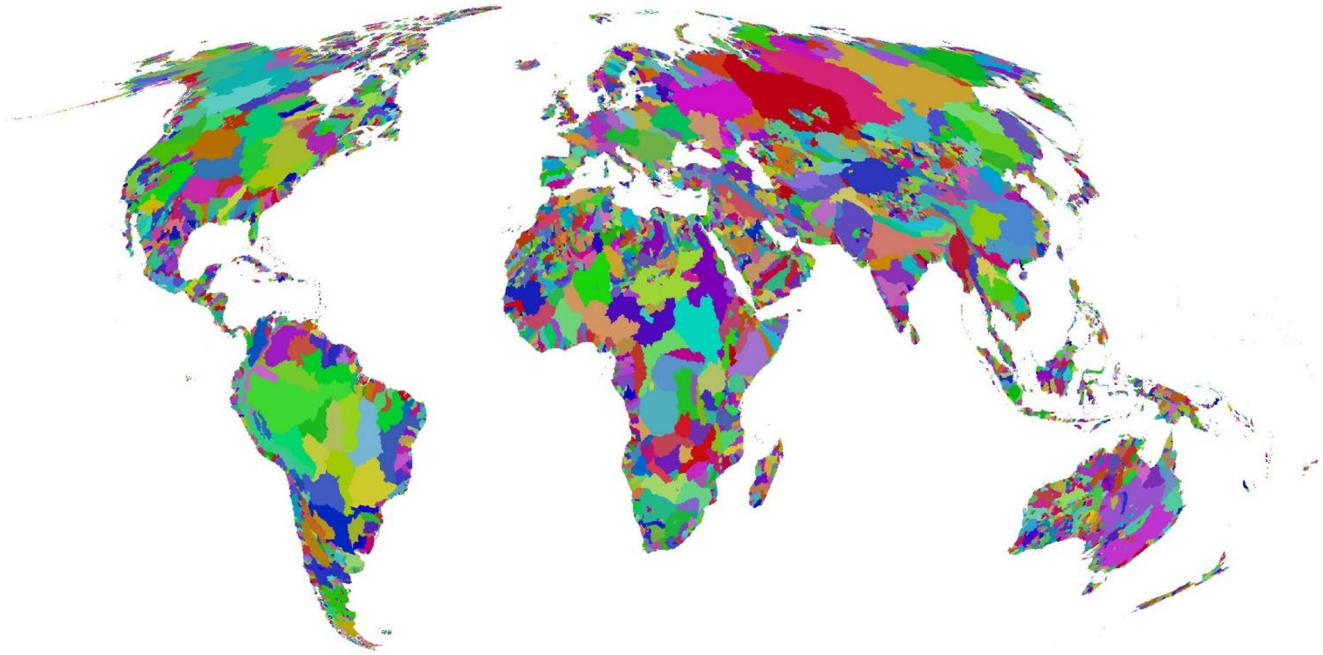

Supplementary Figure 11. Sub-watershed units used for the dispersal scenario. The units were drawn by intersecting the physical watershed boundaries of PCR-GLOBWB's hydrography at 5 arcminutes, with the freshwater ecoregions of the world[10]. In total, 6,525 units are represented (area: μ = 20,376 km^2, σ = 90,717 km^2) from 10,884 main hydrologic basins and 449 freshwater ecoregions.

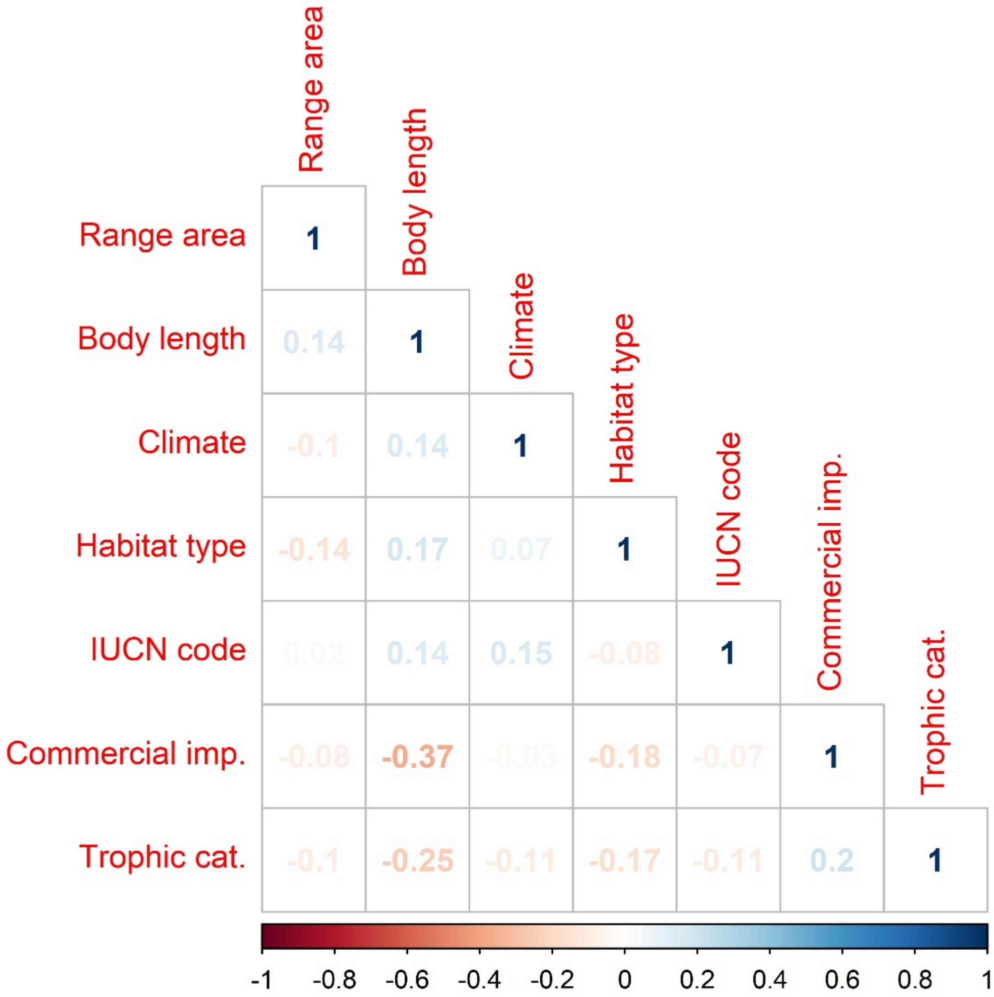

Supplementary Figure 12. Correlation matrix for the explanatory variables used in the phylogenetic regression. Values report the Spearman's correlation coefficient. Variance Inflation Factors (VIFs) for the same set of explanatory variables are reported in Supplementary Table 6.

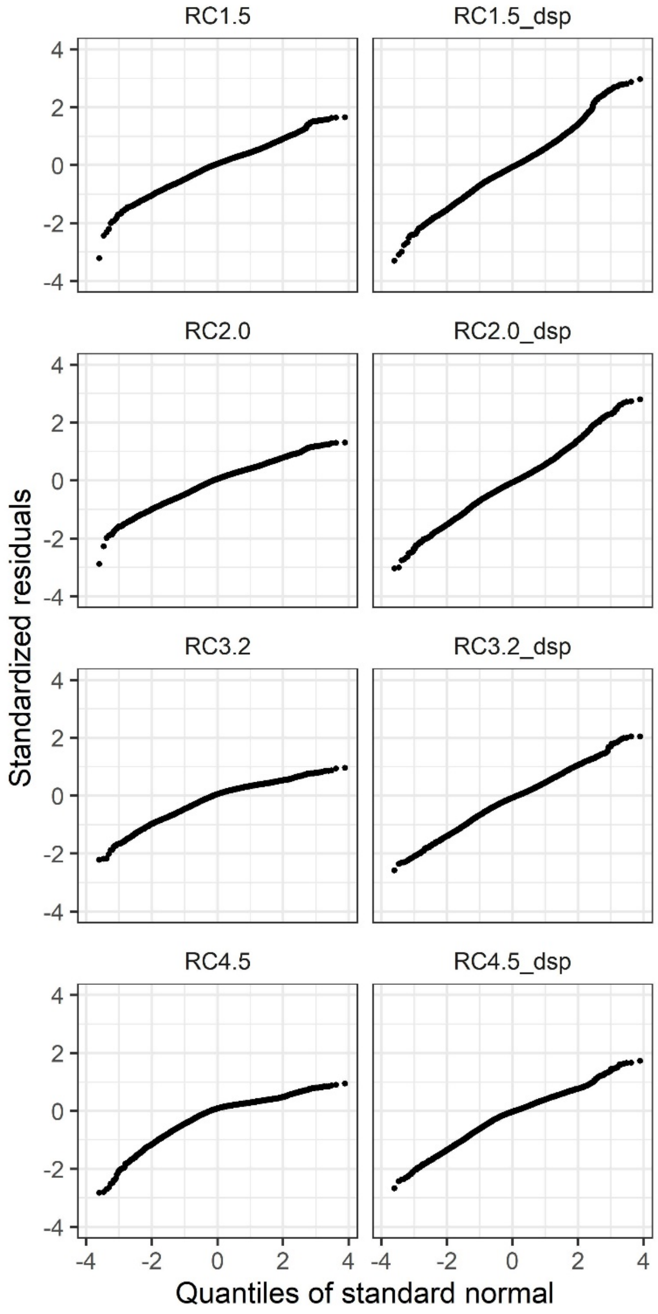

Supplementary Figure 13. Q-Q plots of the phylogenetic regression residuals (average across the 100 replicates based on the stochastically-generated phylogenetic trees) reported for each combination of dispersal scenario (maximal dispersal flagged as "_dsp") and warming level.

Supplementary Tables

Supplementary Table 1. Years at which each GCM-RCP scenario reaches one of the four global warming levels. The levels are defined by global mean temperature rises compared to pre-industrial (1850-1900). Years represent the central value of the 30-years moving average. Three scenarios do not report values as they do not reach even the 1.5^{o}C warming level.

	1.5^{O}C	2 ^{O}C	3.2 ^{O}C	4.5 ^{O}C
**gfdl_rcp2p6**				
**gfdl_rcp4p5**	2044			
**gfdl_rcp6p0**	2052	2073		
**gfdl_rcp8p5**	2034	2051		
**hadgem_rcp2p6**	2028			
**hadgem_rcp4p5**	2029	2044		
**hadgem_rcp6p0**	2034	2048	2082	
**hadgem_rcp8p5**	2024	2036	2058	2080
**ipsl_rcp2p6**	2011	2033		
**ipsl_rcp4p5**	2012	2031		
**ipsl_rcp6p0**	2012	2032	2078	
**ipsl_rcp8p5**	2011	2026	2051	2074
**miroc_rcp2p6**	2018	2035		
**miroc_rcp4p5**	2021	2035		
**miroc_rcp6p0**	2023	2039	2073	
**miroc_rcp8p5**	2018	2030	2053	2074
**noresm_rcp2p6**				
**noresm_rcp4p5**				
**noresm_rcp6p0**	2050	2072		
**noresm_rcp8p5**	2033	2048	2078	

Supplementary Table 2. Abbreviations used for the species order names of Supplementary Figure 6. “Other” groups together order names with less than 20 species available for our analysis.

ORDER NAME	ABBREVIATION USED
Characiformes	Characi.
Pleuronectiformes	Pleuron.
Gymnotiformes	Gymnoti.
Osteoglossiformes	Osteogl.
Siluriformes	Silurif.
Batrachoidiformes	Other
Beloniformes	Belonif.
Perciformes	Percifo.
Clupeiformes	Clupeif.
Cyprinodontiformes	Cyprino.
Lepidosireniformes	Other
Myliobatiformes	Mylioba.
Synbranchiformes	Synbran.
Tetraodontiformes	Tetraod.
Syngnathiformes	Syngnat.
Cypriniformes	Cyprini.
Salmoniformes	Salmoni.
Gonorynchiformes	Gonoryn.
Scorpaeniformes	Scorpae.
Atheriniformes	Atherin.
Mugiliformes	Mugilif.
Gasterosteiformes	Other
Osmeriformes	Osmerif.
Anguilliformes	Anguill.
Acipenseriformes	Other
Percopsiformes	Other
Gadiformes	Other
Polypteriformes	Other
Esociformes	Other
Elopiformes	Other
Lepisosteiformes	Other
Gobiesociformes	Other
Ophidiiformes	Other
Albuliformes	Other
Beryciformes	Other
Rhinopristiformes	Other
Carcharhiniformes	Other
Aulopiformes	Other
Lophiiformes	Other
Petromyzontiformes	Petromy.
Zeiformes	Other

Rajiformes	Other
Myctophiformes	Other
Torpediniformes	Other
Squaliformes	Other
Ateleopodiformes	Other
Orectolobiformes	Other
Ceratodontiformes	Other
Amiiformes	Other
Heterodontiformes	Other

Supplementary Table 3. Mean percentage of geographic range threatened by future climate exposure across the species. Values report the mean across the RCP-GCM scenarios as well as the standard deviation (in brackets) for each warming target and across the different variables. Q = all flow variables, T = all water temperature variables.

Warming level	All	Q	T	Both Q&T	Qmin	Qzf	Qmax	Tmax	Tmin
				**No dispersal**					
**1.5ºC**	13.8 (3.97)	2.9 (0.33)	11.98 (4.04)	1.08 (0.36)	0.71 (0.27)	1.09 (0.28)	1.41 (0.25)	11.88 (4.07)	0.11 (0.05)
**2.0ºC**	21.07 (4.89)	3.49 (0.4)	19.27 (5.01)	1.69 (0.38)	0.83 (0.25)	1.45 (0.3)	1.61 (0.28)	19.19 (5.04)	0.1 (0.07)
**3.2ºC**	42.2 (6.68)	4.72 (0.56)	40.7 (6.93)	3.21 (0.54)	1.2 (0.38)	2.56 (0.51)	1.62 (0.45)	40.66 (6.94)	0.05 (0.02)
**4.5ºC**	61.15 (6.54)	7.26 (1.14)	59.72 (6.65)	5.84 (1.21)	1.87 (1.02)	4.69 (1.2)	1.93 (0.57)	59.7 (6.65)	0.06 (0.05)
				**Maximal dispersal**					
**1.5ºC**	3.15 (1.06)	0.65 (0.06)	2.72 (1.07)	0.28 (0.1)	0.21 (0.12)	0.19 (0.06)	0.33 (0.1)	2.7 (1.08)	0.02 (0.01)
**2.0ºC**	5.47 (1.71)	0.76 (0.07)	5.01 (1.72)	0.41 (0.08)	0.22 (0.11)	0.27 (0.06)	0.37 (0.09)	4.99 (1.72)	0.02 (0.01)
**3.2ºC**	15.86 (4.55)	1 (0.1)	15.29 (4.6)	0.68 (0.1)	0.3 (0.12)	0.5 (0.09)	0.36 (0.12)	15.28 (4.6)	0.01 (0)
**4.5ºC**	31.28 (8.98)	1.6 (0.21)	30.49 (9)	1.27 (0.23)	0.44 (0.25)	0.98 (0.18)	0.44 (0.13)	30.48 (9)	0.01 (0.01)

Supplementary Table 4. Percentage of species with more than half of their geographic range threatened by future climate exposure. Values report the mean across the RCP-GCM scenarios as well as the standard deviation (in brackets) for each warming target and across the different variables. Q = all flow variables, T = all water temperature variables.

Warming level	All	Q	T	Both Q&T	Qmin	Qzf	Qmax	Tmax	Tmin
				**No dispersal**					
**1.5ºC**	4.4 (2.29)	0.76 (0.09)	3.57 (2.25)	0.38 (0.13)	0.23 (0.16)	0.13 (0.06)	0.52 (0.21)	3.56 (2.25)	0 (0.01)
**2.0ºC**	9.42 (4.17)	0.87 (0.11)	8.33 (4.06)	0.57 (0.1)	0.23 (0.14)	0.19 (0.08)	0.59 (0.2)	8.3 (4.07)	0 (0)
**3.2ºC**	35.6 (11.43)	1.07 (0.12)	34.22 (11.65)	0.79 (0.09)	0.33 (0.15)	0.42 (0.1)	0.49 (0.23)	34.19 (11.64)	0 (0)
**4.5ºC**	62.53 (7.47)	1.83 (0.19)	60.91 (7.43)	1.48 (0.17)	0.57 (0.26)	0.98 (0.09)	0.59 (0.18)	60.89 (7.43)	0 (0)
				**Maximal dispersal**					
**1.5ºC**	0.59 (0.21)	0.31 (0.01)	0.44 (0.27)	0.19 (0.11)	0.13 (0.12)	0.02 (0.02)	0.22 (0.11)	0.44 (0.27)	0 (0.01)
**2.0ºC**	1.4 (0.71)	0.33 (0.02)	1.23 (0.71)	0.25 (0.07)	0.11 (0.11)	0.04 (0.02)	0.25 (0.08)	1.23 (0.71)	0 (0)
**3.2ºC**	7.5 (3.46)	0.36 (0.06)	7.17 (3.45)	0.28 (0.06)	0.15 (0.11)	0.09 (0.05)	0.21 (0.1)	7.17 (3.45)	0 (0)
**4.5ºC**	23.59 (12.83)	0.52 (0.1)	22.84 (12.88)	0.4 (0.05)	0.18 (0.12)	0.18 (0.11)	0.27 (0.02)	22.83 (12.88)	0 (0)

Supplementary Table 5-I. Coefficients of the phylogenetic regression at different warming levels under no dispersal assumption. Body length and range area are continuous variables and were log-transformed. For the remaining categorical variables, habitat = habitat type and differentiate species that are found only in streams/rivers (lotic) from those that are found also in lakes (lentic) and in the marine environment. Climate zone refers to the Köppen-Geiger climate zones, where A=Equatorial (reference level), B=Arid, C=Warm temperate, D=Snow, E=Polar. IUCN code refers to the IUCN threat status, where CR=Critically endangered (reference level), VU=Vulnerable, NT=Near Threatened, LC=Least Concern, EN=Endangered, DD=Data Deficient. Commercial refers to commercial relevance, where com. = "of commercial importance" (reference level), NoInt. = "of no interest", Subs. = "subsistence fisheries" (DD = data deficient)[11]. Trophic refers to the trophic level, categorized in Carnivorous (Carn.) (reference level), Omnivorous (Omni.) and Herbivorous (Herbi.) species (DD = data deficient). The first category of the categorical variables (flagged as reference level) not showing among the variables in the table is embedded in the intercept.

variable	1.5°C (λ = 0.97, r = 0.47)			2.0°C (λ = 0.96, r = 0.50)			3.2°C (λ = 0.96, r = 0.55)			4.5°C (λ = 0.96, r = 0.59)			Mean coef	St. dev. Coef
	coef	|t|-stat	p-val	coef	|t|-stat	p-val	coef	|t|-stat	p-val	coef	|t|-stat	p-val		
**(Intercept)**	1.89	9.29	<0.001	1.94	8.81	<0.001	1.96	8.20	<0.001	1.99	10.82	<0.001	1.95	0.04
**Range area**	-0.14	34.61	<0.001	-0.12	28.64	<0.001	-0.06	14.75	<0.001	-0.03	9.76	<0.001	-0.09	0.04
**Body length**	-0.07	6.30	<0.001	-0.07	6.60	<0.001	-0.07	7.19	<0.001	-0.05	6.19	<0.001	-0.07	0.01
**habitat_Lotic**	0.00	0.24	0.805	0.01	1.16	0.251	0.01	1.79	0.078	0.01	0.87	0.392	0.01	0.00
**habitat_Marine**	-0.26	17.28	<0.001	-0.25	16.65	<0.001	-0.23	15.87	<0.001	-0.20	16.65	<0.001	-0.23	0.03
**climate_zone_B**	0.11	7.70	<0.001	0.11	7.88	<0.001	0.08	5.82	<0.001	0.06	5.36	<0.001	0.09	0.02
**climate_zone_C**	-0.01	1.49	0.142	-0.01	0.68	0.502	-0.02	2.52	0.013	-0.03	4.65	<0.001	-0.02	0.01
**climate_zone_D**	0.04	1.46	0.150	0.03	1.36	0.179	0.01	0.45	0.655	-0.04	1.89	0.063	0.01	0.03
**climate_zone_E**	-0.18	2.55	0.012	-0.18	2.63	0.010	-0.19	2.97	0.004	-0.25	4.71	<0.001	-0.20	0.03
**IUCN_code_DD**	-0.10	4.17	<0.001	-0.09	3.91	<0.001	-0.08	3.43	<0.001	-0.05	2.85	0.005	-0.08	0.02
**IUCN_code_EN**	-0.10	3.58	<0.001	-0.08	3.17	0.002	-0.06	2.32	0.021	-0.03	1.58	0.116	-0.07	0.02
**IUCN_code_LC**	-0.17	6.89	<0.001	-0.17	6.93	<0.001	-0.15	6.57	<0.001	-0.10	5.57	<0.001	-0.15	0.03
**IUCN_code_NT**	-0.14	4.94	<0.001	-0.14	4.86	<0.001	-0.10	3.82	<0.001	-0.07	3.05	0.002	-0.11	0.03
**IUCN_code_VU**	-0.10	3.73	<0.001	-0.09	3.47	<0.001	-0.07	2.87	0.004	-0.04	2.17	0.032	-0.08	0.02
**commercial_DD**	0.03	2.55	0.011	0.04	3.74	<0.001	0.06	5.87	<0.001	0.06	7.09	<0.001	0.05	0.01
**commercial_NoInt.**	-0.04	2.41	0.017	-0.03	2.03	0.044	-0.02	1.28	0.203	0.00	0.22	0.825	-0.02	0.01
**commercial_Subs.**	0.01	0.49	0.628	0.01	0.34	0.732	0.00	0.12	0.888	0.01	0.61	0.542	0.01	0.00
**trophic_DD**	0.01	1.37	0.174	0.02	2.22	0.027	0.03	3.66	<0.001	0.03	4.39	<0.001	0.02	0.01
**trophic_Herbi.**	-0.03	1.37	0.175	-0.02	0.99	0.323	-0.01	0.35	0.726	0.00	0.29	0.776	-0.01	0.01
**trophic_Omni.**	-0.02	1.01	0.318	-0.02	0.90	0.370	-0.01	0.70	0.485	-0.01	0.75	0.457	-0.01	0.00

Supplementary Table 5-II. Coefficients of the phylogenetic regression at different warming levels under maximal dispersal assumption. Body length and range area are continuous variables and were log-transformed. For the remaining categorical variables, habitat = habitat type and differentiate species that are found only in streams/rivers (lotic) from those that are found also in lakes (lentic) and in the marine environment. Climate zone refers to the Köppen-Geiger climate zones, where A=Equatorial (reference level), B=Arid, C=Warm temperate, D=Snow, E=Polar. IUCN code refers to the IUCN threat status, where CR=Critically endangered (reference level), VU=Vulnerable, NT=Near Threatened, LC=Least Concern, EN=Endangered, DD=Data Deficient. Commercial refers to commercial relevance, where com. = "of commercial importance" (reference level), NoInt. = "of no interest", Subs. = "subsistence fisheries" (DD = data deficient)[11]. Trophic refers to the trophic level, categorized in Carnivorous (Carn.) (reference level), Omnivorous (Omni.) and Herbivorous (Herbi.) species (DD = data deficient). The first category of the categorical variables (flagged as reference level) not showing among the variables in the table is embedded in the intercept.

variable	1.5°C (λ = 0.94, r = 0.47)			2.0°C (λ = 0.91, r = 0.49)			3.2°C (λ = 0.94, r = 0.52)			4.5°C (λ = 0.95, r = 0.55)			Mean coef	St. dev. Coef
	coef	|t|-stat	p-val	coef	|t|-stat	p-val	coef	|t|-stat	p-val	coef	|t|-stat	p-val		
**(Intercept)**	-0.25	1.28	0.201	-0.05	0.26	0.797	0.38	1.44	0.150	0.89	3.12	0.002	0.24	0.44
**Range area**	0.16	22.97	<0.001	0.17	23.77	<0.001	0.19	25.82	<0.001	0.16	23.50	<0.001	0.17	0.01
**Body length**	-0.12	6.88	<0.001	-0.14	7.52	<0.001	-0.18	9.42	<0.001	-0.19	10.67	<0.001	-0.16	0.03
**habitat_Lotic**	0.05	3.51	<0.001	0.06	3.98	<0.001	0.05	3.34	<0.001	0.02	1.31	0.194	0.05	0.02
**habitat_Marine**	-0.42	16.62	<0.001	-0.42	16.30	<0.001	-0.42	15.89	<0.001	-0.42	17.10	<0.001	-0.42	0.00
**climate_zone_B**	0.01	0.44	0.660	0.00	0.13	0.895	-0.06	2.45	0.015	-0.11	4.64	<0.001	-0.04	0.05
**climate_zone_C**	-0.08	5.74	<0.001	-0.08	5.22	<0.001	-0.11	7.34	<0.001	-0.14	10.32	<0.001	-0.10	0.03
**climate_zone_D**	-0.09	2.22	0.028	-0.08	1.97	0.051	-0.09	2.14	0.035	-0.15	3.76	<0.001	-0.10	0.03
**climate_zone_E**	-0.11	0.91	0.364	-0.13	1.06	0.293	-0.20	1.65	0.103	-0.32	2.87	0.005	-0.19	0.09
**IUCN_code_DD**	-0.29	6.93	<0.001	-0.28	6.64	<0.001	-0.26	5.92	<0.001	-0.20	5.14	<0.001	-0.26	0.03
**IUCN_code_EN**	-0.01	0.11	0.902	0.00	0.03	0.940	0.01	0.11	0.901	0.01	0.18	0.853	0.00	0.00
**IUCN_code_LC**	-0.30	7.16	<0.001	-0.31	7.27	<0.001	-0.32	7.44	<0.001	-0.29	7.27	<0.001	-0.30	0.01
**IUCN_code_NT**	-0.20	4.17	<0.001	-0.21	4.30	<0.001	-0.21	4.23	<0.001	-0.20	4.26	<0.001	-0.21	0.01
**IUCN_code_VU**	-0.09	2.02	0.044	-0.09	1.92	0.056	-0.08	1.60	0.111	-0.06	1.44	0.153	-0.08	0.01
**commercial_DD**	0.11	5.47	<0.001	0.12	6.12	<0.001	0.15	7.55	<0.001	0.16	8.84	<0.001	0.14	0.02
**commercial_NoInt.**	0.02	0.66	0.512	0.02	0.59	0.556	0.01	0.54	0.592	0.02	0.86	0.393	0.02	0.00
**commercial_Subs.**	0.08	1.97	0.049	0.07	1.72	0.087	0.05	1.08	0.280	0.05	1.17	0.242	0.06	0.02
**trophic_DD**	0.03	1.92	0.055	0.04	2.57	0.010	0.06	3.94	<0.001	0.07	4.61	<0.001	0.05	0.02
**trophic_Herbi.**	-0.02	0.52	0.605	-0.01	0.33	0.741	0.00	0.06	0.924	-0.01	0.31	0.761	-0.01	0.01
**trophic_Omni.**	0.03	1.02	0.308	0.03	0.97	0.334	0.02	0.76	0.448	0.02	0.66	0.511	0.03	0.00

Supplementary Table 6. Variance Inflation Factors (VIFs) among the explanatory variables used in the phylogenetic regression.

Variable	VIF
Range area	1.07
Body length	1.25
Climate	1.06
Habitat type	1.11
IUCN code	1.05
Commercial imp.	1.14
Trophic cat.	1.06

Supplementary References

1. Tedesco, P. A. *et al.* Data Descriptor: A global database on freshwater fish species occurrence in drainage basins. *Sci. Data* **4**, 1–6 (2017).

2. Comte, L. & Olden, J. D. Climatic vulnerability of the world's freshwater and marine fishes. *Nat. Clim. Chang.* **7**, 718–722 (2017).

3. Bennett, J. M. *et al.* GlobTherm, a global database on thermal tolerances for aquatic and terrestrial organisms. *Sci. Data* **5**, 180022 (2018).

4. Warszawski, L. *et al.* The Inter-Sectoral Impact Model Intercomparison Project (ISI–MIP): Project framework. *Proc. Natl. Acad. Sci.* **111**, 3228–3232 (2014).

5. Schewe, J. *et al.* Multimodel assessment of water scarcity under climate change. *Proc. Natl. Acad. Sci.* **111**, 3245–3250 (2014).

6. Hempel, S., Frieler, K., Warszawski, L., Schewe, J. & Piontek, F. A trend-preserving bias correction – the ISI-MIP approach. *Earth Syst. Dyn.* **4**, 219–236 (2013).

7. Sutanudjaja, E. H. *et al.* PCR-GLOBWB 2: a 5 arcmin global hydrological and water resources model. *Geosci. Model Dev.* **11**, 2429–2453 (2018).

8. Wanders, N., van Vliet, M. T. H., Wada, Y., Bierkens, M. F. P. & van Beek, L. P. H. High-resolution global water temperature modelling. *Water Resour. Res.* (2019) doi:10.1029/2018WR023250.

9. Allen, R. G., Pereira, L. S., Raes, D. & Smith, M. Crop evapotranspiration-Guidelines for computing crop water requirements-FAO Irrigation and drainage paper 56. *Fao, Rome* **300**, D05109 (1998).

10. Abell, R. *et al.* Freshwater Ecoregions of the World: A New Map of Biogeographic Units for Freshwater Biodiversity Conservation. *Bioscience* **58**, 403–414 (2008).

11. Froese, R. & Pauly, D. FishBase. *World Wide Web electronic publication* www.fishbase.org (2018).
