# Supplementary material for: Threats of global warming to the world’s freshwater fishes
Source: Nat Commun. 2021 Mar 15;12:1701. doi: 10.1038/s41467-021-21655-w (PMC7960982; doi:10.1038/s41467-021-21655-w)
Supplement: Supplementary file 3 — Description of Additional Supplementary Files [file 41467_2021_21655_MOESM3_ESM.pdf]

Descriptions of Additional Supplementary Files

Supplementary Data 1. Range Threatened (RT) in % at different warming levels (1.5^{o}C, 2.0^{o}C, 3.2^{o}C, 4.5^{o}C) and under maximal and no dispersal assumptions for each of the 11,425 species assessed (Figure 1). Data are provided as a table in CSV format.

Supplementary Data 2. Potentially Affected Fraction (PAF; see Methods section of the main manuscript for the definition) raster layers at 5 arcmin resolution at different warming levels (1.5^{o}C, 2.0^{o}C, 3.2^{o}C, 4.5^{o}C) for the no dispersal scenario. PAF raster layers are provided in GeoTIFF format and WGS84 coordinate reference system. PAF maps are available based on all variables (no suffix; Figure 2) as well as on single variables (suffix specifies variable; Figure 4, Supplementary Figures 2 and 3). Qma: maximum weekly flow; Qmi: minimum weekly flow; Qzf: number of zero flow weeks; Tma: maximum weekly water temperature; Tmi: minimum weekly water temperature; Q_all: all streamflow variables; T_all: all water temperature variables; both_QT: overlap of all flow and water temperature variables.

Supplementary Data 3. Potentially Affected Fraction (PAF; see Methods section of the main manuscript for the definition) raster layers at 5 arcmin resolution at different warming levels (1.5^{o}C, 2.0^{o}C, 3.2^{o}C, 4.5^{o}C) for the maximal dispersal scenario. PAF raster layers are provided in GeoTIFF format and WGS84 coordinate reference system. PAF maps are available based on all variables (no suffix; Figure 2) as well as on single variables (suffix specifies variable; Figure 4, Supplementary Figure 2 and 3). Qma: maximum weekly flow; Qmi: minimum weekly flow; Qzf: number of zero flow weeks; Tma: maximum weekly water temperature; Tmi: minimum weekly water temperature; Q_all: all streamflow variables; T_all: all water temperature variables; both_QT: overlap of all flow and water temperature variables.

Supplementary Data 4. Potentially Affected Fraction (PAF; see Methods section of the main manuscript for the definition) averaged over the basin area for 200 main hydrologic basins (defined as having an outlet to the sea/ocean). Next to each basin name the number of species assessed by this study are reported in brackets. Average PAFs are reported at different warming levels (1.5^{o}C, 2.0^{o}C, 3.2^{o}C, 4.5^{o}C) and under maximal and no dispersal assumptions (Figure 3 and Supplementary Figure 4). Data are provided as a table in CSV format.

Supplementary Data 5. Variable importance stemming from the phylogenetic regression on species traits (Figure 5). Variable importance is reported at each warming level (1.5^{o}C, 2.0^{o}C, 3.2^{o}C, 4.5^{o}C) and dispersal assumptions (suffix "dsp" denotes the maximal dispersal assumption, no suffix stands for the no dispersal assumption). Mean and standard deviation across the 100 replicates based on stochastically generated phylogenetic trees are reported. Data are provided as a table in CSV format.

Supplementary Data 6. Initial Species Richness (SR) based on the geographic ranges of the 11,425 species analyzed in this study (Supplementary Figure 9). SR is reported as the total number of species occurring on a 5 arcminutes grid-cell. The SR raster layer is provided in GeoTIFF format and WGS84 coordinate reference system.
