# Supplementary material for: Threats of global warming to the world’s freshwater fishes
Source: Nat Commun. 2021 Mar 15;12:1701. doi: 10.1038/s41467-021-21655-w (PMC7960982; doi:10.1038/s41467-021-21655-w)
Supplement: Supplementary file 10 — Reporting Summary [file 41467_2021_21655_MOESM10_ESM.pdf]

nature research

Corresponding author(s): Valerio Barbarossa

Last updated by author(s): Dec 8, 2020

Reporting Summary

Nature Research wishes to improve the reproducibility of the work that we publish. This form provides structure for consistency and transparency in reporting. For further information on Nature Research policies, see Authors & Referees and the Editorial Policy Checklist .

Statistics

For all statistical analyses, confirm that the following items are present in the figure legend, table legend, main text, or Methods section.

n/a	Confirmed	
☐	☒	The exact sample size (n) for each experimental group/condition, given as a discrete number and unit of measurement
☐	☒	A statement on whether measurements were taken from distinct samples or whether the same sample was measured repeatedly
☐	☒	The statistical test(s) used AND whether they are one- or two-sided<br>*Only common tests should be described solely by name; describe more complex techniques in the Methods section.*
☐	☒	A description of all covariates tested
☒	☐	A description of any assumptions or corrections, such as tests of normality and adjustment for multiple comparisons
☐	☒	A full description of the statistical parameters including central tendency (e.g. means) or other basic estimates (e.g. regression coefficient) AND variation (e.g. standard deviation) or associated estimates of uncertainty (e.g. confidence intervals)
☐	☒	For null hypothesis testing, the test statistic (e.g. F, t, r) with confidence intervals, effect sizes, degrees of freedom and P value noted<br>*Give P values as exact values whenever suitable.*
☒	☐	For Bayesian analysis, information on the choice of priors and Markov chain Monte Carlo settings
☒	☐	For hierarchical and complex designs, identification of the appropriate level for tests and full reporting of outcomes
☐	☒	Estimates of effect sizes (e.g. Cohen's d, Pearson's r), indicating how they were calculated

Our web collection on statistics for biologists contains articles on many of the points above.

Software and code

Policy information about availability of computer code

Data collection	Fish species traits data were retrieved from IUCN and Fishbase through API services in R version 3.6.2 ("rfishbase" and "rredlist" packages). Weekly streamflow and water temperature for different scenarios in the period 1960-2100 were obtained from the hydrological model PCR-GLOBWB (code available at https://github.com/UU-Hydro/PCR-GLOBWB_model) coupled to the water temperature model dynWat (code available at https://github.com/wande001/dynWat). Meteorological input data for the PCR-GLOBWB model was downloaded from https://www.isimip.org/. All the PCR-GLOBWB-dynWat runs were carried out on the Dutch national e-infrastructure Cartesius.
Data analysis	The R code used for the quantification of percentage of range threatened is available at https://github.com/vbarbarossa/fishsuit. Phylogenetic regression was performed using R version 3.6.2 and packages "fishtree" and "nlme." All the model runs were carried out on the Dutch national e-infrastructure Cartesius.

For manuscripts utilizing custom algorithms or software that are central to the research but not yet described in published literature, software must be made available to editors/reviewers. We strongly encourage code deposition in a community repository (e.g. GitHub). See the Nature Research guidelines for submitting code & software for further information.

Data

Policy information about availability of data

All manuscripts must include a data availability statement. This statement should provide the following information, where applicable:

- Accession codes, unique identifiers, or web links for publicly available datasets
- A list of figures that have associated raw data
- A description of any restrictions on data availability

Data associated with this publication including source data files for Figures 1 to 5 of this manuscript are available within the paper and its supplementary information files. Species' geographical ranges were downloaded from the IUCN repository https://www.iucnredlist.org/resources/spatial-data-download, from Jezequel et al., 2020 (https://www.nature.com/articles/s41597-020-0436-4) and a combination of additional sources as described in Barbarossa et al., 2020 (https://www.pnas.org/content/117/7/3648).

Field-specific reporting

Please select the one below that is the best fit for your research. If you are not sure, read the appropriate sections before making your selection.

☐ Life sciences ☐ Behavioural & social sciences ☒ Ecological, evolutionary & environmental sciences

For a reference copy of the document with all sections, see nature.com/documents/nr-reporting-summary-flat.pdf

Ecological, evolutionary & environmental sciences study design

All studies must disclose on these points even when the disclosure is negative.

Study description	Future climate threats to 11,425 riverine fish species quantified as exposure to flow and water temperature extremes under different global warming scenarios
Research sample	Geographical distribution of 11,425 riverine fish species. Riverine fish belon to the following orders: Characiformes, Pleuronectiformes, Gymnotiformes, Osteoglossiformes, Siluriformes, Batrachoidiformes, Beloniformes, Perciformes, Clupeiformes, Cyprinodontiformes, Lepidosireniformes, Myliobatiformes, Synbranchiformes, Tetraodontiformes, Syngnathiformes, Cypriniformes, Salmoniformes, Gonorynchiformes, Scorpaeniformes, Atheriniformes, Mugiliformes, Gasterosteiformes, Osmeriformes, Anguilliformes, Acipenseriformes, Percopsiformes, Gadiformes, Polypteriformes, Esociformes, Elopiformes, Lepisosteiformes, Gobiesociformes, Ophidiiformes, Albuliformes, Beryciformes, Rhinopristiformes, Carcharhiniformes, Aulopiformes, Lophiiformes, Petromyzontiformes, Zeiformes, Rajiformes, Myctophiformes, Torpediniformes, Squaliformes, Ateleopodiformes, Orectolobiformes, Ceratodontiformes, Amiiformes, Heterodontiformes. Species' geographical ranges were downloaded from the IUCN repository https://www.iucnredlist.org/resources/spatial-data-download, from Jezequel et al., 2020 (https://www.nature.com/articles/s41597-020-0436-4) and a combination of additional sources as described in Barbarossa et al., 2020 (https://www.pnas.org/content/117/7/3648).
Sampling strategy	We resampled the range polygons of each species to the 5 arcminutes (~10 km) hydrography of the global hydrological model, with a given species marked as occurring in a cell if >= 50% of the cell area overlapped with the species' polygon. In total, we obtained geographic ranges for 12,934 freshwater fish species (~90% of the known freshwater fish species). We excluded 1,160 exclusively lentic species because our hydrological model is less adequate for lakes than for rivers, i.e., it does not account for water temperature stratification. Out of the 11,774 (partially or entirely) lotic fish species, we excluded 349 species (~3%) because their occurrence range was smaller than ~1,000 km2 (i.e., 10 grid cells), which we considered too small relative to the spatial resolution of the hydrological model.
Data collection	Species' geographical ranges were downloaded from the IUCN repository https://www.iucnredlist.org/resources/spatial-data-download, rom Jezequel et al., 2020 (https://www.nature.com/articles/s41597-020-0436-4) and a combination of additional sources as described in Barbarossa et al., 2020 (https://www.pnas.org/content/117/7/3648). Fish species traits data were retrieved from IUCN and Fishbase through API services in R. Weekly streamflow and water temperature for different scenarios in the period 1960-2100 were obtained from the hydrological model PCR-GLOBWB (python code available at https://github.com/UU-Hydro/PCR-GLOBWB_model) coupled to the water temperature model dynWat (python code available at https://github.com/wande001/dynWat). Meteorological input data for the PCR-GLOBWB model was downloaded from https://www.isimip.org/.
Timing and spatial scale	Global extent (excluding Antarctica). Weekly flow and water temperature were simulated from 1976 to 2100 under five GCMs and four RCPs (future RCP projections start at year 2006) scenarios.
Data exclusions	Prior to running the model, we excluded 1,160 exclusively lentic species because our hydrological model is less adequate for lakes than for rivers, i.e., it does not account for water temperature stratification. Out of the 11,774 (partially or entirely) lotic fish species, we excluded 349 species (~3%) because their occurrence range was smaller than ~1,000 km2 (i.e., 10 grid cells), which we considered too small relative to the spatial resolution of the hydrological model.
Reproducibility	The reproducibility of the framework to calculate species-specific percentage of range threatened has been tested by multiple runs where different parameters of the model were adjusted. The code written for the numerical simulations was tested only on a linux environment and its reproducibility on a different OS was not tested. All the model runs were successful.
Randomization	100-fold randomizations were performed for each covariate of the phylogenetic regression to test for variable importance

Blinding	Not relevant, this study did not perform tests on subjects aware of the experiment.

Did the study involve field work? ☐ Yes ☒ No

Reporting for specific materials, systems and methods

We require information from authors about some types of materials, experimental systems and methods used in many studies. Here, indicate whether each material, system or method listed is relevant to your study. If you are not sure if a list item applies to your research, read the appropriate section before selecting a response.

Materials & experimental systems

n/a	Involved in the study	
☒	☐	Antibodies
☒	☐	Eukaryotic cell lines
☒	☐	Palaeontology
☒	☐	Animals and other organisms
☒	☐	Human research participants
☒	☐	Clinical data

Methods

n/a	Involved in the study	
☒	☐	ChIP-seq
☒	☐	Flow cytometry
☒	☐	MRI-based neuroimaging
